# Millimeter wave gesture recognition using multi-feature fusion models in complex scenes

**DOI:** 10.1038/s41598-024-64576-6

**Published:** 2024-06-14

**Authors:** Zhanjun Hao, Zhizhou Sun, Fenfang Li, Ruidong Wang, Jianxiang Peng

**Affiliations:** 1 College of Computer Science and Engineering, Northwest Normal University, Lanzhou, 730070 China; 2 Gansu Province Internet of Things Engineering Research Centre, Northwest Normal University, Lanzhou, 730070 China

**Keywords:** Mathematics and computing, Computer science

## Abstract

As a form of body language, the gesture plays an important role in smart homes, game interactions, and sign language communication, etc. The gesture recognition methods have been carried out extensively. The existing methods have inherent limitations regarding user experience, visual environment, and recognition granularity. Millimeter wave radar provides an effective method for the problems lie ahead gesture recognition because of the advantage of considerable bandwidth and high precision perception. Interfering factors and the complexity of the model raise an enormous challenge to the practical application of gesture recognition methods as the millimeter wave radar is applied to complex scenes. Based on multi-feature fusion, a gesture recognition method for complex scenes is proposed in this work. We collected data in variety places to improve sample reliability, filtered clutters to improve the signal’s signal-to-noise ratio (SNR), and then obtained multi features involves range-time map (RTM), Doppler-time map (DTM) and angle-time map (ATM) and fused them to enhance the richness and expression ability of the features. A lightweight neural network model multi-CNN-LSTM is designed to gestures recognition. This model consists of three convolutional neural network (CNN) for three obtained features and one long short-term memory (LSTM) for temporal features. We analyzed the performance and complexity of the model and verified the effectiveness of feature extraction. Numerous experiments have shown that this method has generalization ability, adaptability, and high robustness in complex scenarios. The recognition accuracy of 14 experimental gestures reached 97.28%.

## Introduction

Gestures are physical movements that people use fingers, hands, arms, head, face, or body to convey information and interact with the environment^1^, they play a crucial role in human communications. The information is relatively peculiar which is transmitted and communicated through the forms of gestures. So, it is difficult to recognize the meaning of gestures, and required superior specialized knowledge. In order to understand the meaning of gestures exactly, auxiliary technologies for gesture recognition have been studied extensively in recent years, and applied to variety scenarios widely such as human–machine interface^2–5^, sign language recognition^6–8^ and smart home^9–12^, etc. Various traditional methods of gesture recognition are proposed based on different technologies, such as wearable-based device^13–16^ by deploying sensing gloves and sensors on the arms, visualization-based methods^17–19^ relying on the images, such as camera^20–22^, infrared imaging, etc. The traditional methods have the inherent defects in terms of user experience, environment limitation and privacy protection.

For the advantage of wireless perception such as convenient and non-interference, non- visual environment limitation and information protection etc., researchers have conducted extensive research on wireless perception gesture recognition. Acoustic wave is a common mechanical wave which is used in gesture recognition research^23,24^. As a ubiquitous wireless technology, Wi-Fi-based gesture recognition have been widely conducted^25–28^. As a perception technology, Radio Frequency (RF) radar has a fine perception granularity for the sake of its high-frequency and wide-bandwidth. The perception based on radar is completed by obtaining the motion parameters such as the speed, distance, and angle of the target object. Gesture perception with RF radar mainly includes pulse-radar-based^29^ and Continuous Wave (CW) based radar^30^. Long detection distance but slow scanning speed of pulse radar poses a disadvantage in perceiving fast moving targets. CW radar emits continuous wave signals at a constant frequency, which can obtain Doppler frequency shift through echo signals to determine target velocity. Determining the motion state of objects based on velocity alone cannot meet the needs of high accuracy. More parameters of target motion such as distance, speed, and angle should be captured by Frequency-Modulated Continuous Wave (FMCW) radar via fixed slope frequency modulated signals. Therefore, FMCW-based gestures recognition has conducted widespread thorough research^31–35^. With the rapid development of machine learning and deep learning, machine learning algorithms and neural network models have been applied to gesture recognition researches. Machine learning algorithms such as Support Vector Machine (SVM) and k-Nearest Neighbor (KNN) complete the classification and recognition of gesture actions by classifying gesture features^36,37^. In recent years, neural network models have been enjoyed great popularity in gesture recognition researches. Artificial Neural Network (ANN)^38,39^, Recurrent Neural Network (RNN)^40^, LSTM^41,42^ and some other models have been applied to gesture recognition. The CNN generated an excellent result based on visual gesture. Visual-based CNN method is the real optical reflection of limbs, but it doesn’t avoid the defects of visualization-based methods. The motion information parameters of FMCW radar are saved as images, and they are used to recognition by CNN so that avoiding the defects of visualization-based methods^43–46^.

The existing researches on gesture recognition have achieved great results with high accuracy. However, considering the practical value of recognition methods in the complex scenes that approximate real-life scene, the various interference objects provide us numerous challenges. Firstly, how can we improve the recognition model's adaptability and generalization ability in complex scenes. Secondly, as gestures are combined of various macroscopic and subtle actions, the expression ability of signals for gestures is insufficient. We need improve the expression ability to represent gestures more comprehensively. Finally, on account of the resource requirements of models, it is a practical issue to improve model efficiency.

Based on FMCW, this study proposed a gesture recognition method in complex scenes based on multi-feature fusion. By processed the radar signal, we extracted and fused RTM, DTM and ATM of the gestures to improve the expression ability of the signals. The multi-scene data collection generalized the particularity of the scene. A lightweight neural network model Multi-CNN-LSTM is designed to overcome the problems of model efficiency. The main achievements and contributions of this paper are as follows:

1. We design a lightweight gesture recognition model Multi-CNN-LSTM. The model has the advantages of low complexity and high accuracy. It further enhances the practicality of gesture recognition methods.
2. We abstracted multi features and fused them to improve the feature richness. This method fully utilizes the performance advantages of FMCW radar. The distance, speed, angle, and time information of target gestures are expressed more comprehensively, thereby the expression ability of gesture features is improved in complex scenes, so the recognition effectiveness is enhanced.
3. Experimental data is collected in various scenarios. It enhances the richness of data samples in complex scenes, and provides gesture data processing methods in complex scenes. The model's adaptability and generalization ability are further enhanced to complex scenes.

The following chapters are organized as follows: “Related works” section mainly introduced the related work, “System design” section introduced the gesture recognition system proposed in this paper in detail, “Experiment and result analysis” section introduced the experimental process and analyzed the experimental results, and “Conclusion” section take a conclusion of this study.

## Related works

The existing gesture recognition researches have invested a lot of work, which are reviewed in this section. Since our method is implemented through FMCW sensing and lightweight model multi-CNN-LSTM, the work we review focus on the following two topics: gestures sensing method and lightweight model.

### Gestures sensing method

In recent years, there has been a large number of works on gestures sensing methods. We organize state-of-the-art sensing methods in terms of their applications.

#### Wearable-based gesture recognition

Wearable devices utilize sensing gloves, sensors, and other devices deployed on the arm to obtain state information about gesture movement. Pan et al.^47^ showed a hybrid flexible wearable system composed of a simple bimodal capacitive sensor and a customized low-power interface circuit integrated with machine learning algorithm to recognize complex gestures. Ling et al. ^48^ conducted a comparative study on gesture recognition based on accelerometer (ACC) and photoelectric plethysmography (PPG) sensors, and verified that PPG signals are more suitable for gesture interaction on wearable devices. Yuan et al.^49^ designed a type of integrated MXene/polyurethane sensor based on textile fabrics, prepared a data glove, a smart wristband, and a smart elbow pad. And realized a wearable system for gesture recognition by the synergy of those three devices.

#### Visualization-based gesture recognition

Visual channels such as cameras can obtain intuitive gesture motion status. Sharma et al.^50^ used RGB cameras to collect a large number of Indian Sign Language (ISL) gesture sets and designed a CNN model to recognize gestures. An enhanced dense connected convolutional neural network EDenseNet is proposed by Tan et al. for gesture recognition based on vision, and achieved an average accuracy of 98.50%^51^. Wang et al. proposed a vision-based framework consisting of three parts (worker detection and tracking, identification queue equation and gesture recognition), which captures and interprets workers' gestures as human–machine interfaces during construction. The overall accuracy and recall rates were 87.0% and 66.7%, respectively^52^.

#### Wireless sensing-based gesture recognition

The Channel State Information (CSI) and Received Signal Strength Indication (RSSI) of Wi-Fi signals can form perceptual information for gestures used for recognition. Gao et al. established a mathematical model based on Wi-Fi by combining gesture signal and environmental noise, and proposed a signal processing framework with an average accuracy of more than 94%^53^. Gu et al. proposed a Wi-Fi gesture recognition system WiGRENT using a dual attention network. The training network dynamically pays attention to the domain-independent features of gestures on the Wi-Fi channel state information through the spatio-temporal dual attention mechanism^25^. Tang et al. designed a LSTM-FCN model to extract Wi-Fi gesture features from different dimensions, and achieved an average accuracy of about 98.9%^54^. Acoustic wave is a mechanical wave that used the reflection principle of sound wave to obtain the gesture signal for processing, obtain the gesture information, and complete the gesture recognition through the perceived signal. Amesaka et al. used the speaker to send the ultrasonic sweep sine signal, and the microphone simultaneously records the ultrasonic signal transmitted through the clothes and the friction sound generated by the gestures on the clothes, combining active and passive acoustic sensors to recognize various gestures^55^. Wang et al. proposed StruGesture on the back of mobile phones using ultrasonic signals, which uses structural sound to recognize the sliding gesture on the back of mobile phones^56^. Wang et al. designed a system called SignGest to capture users' sign language gestures through a built-in microphone^57^.

By the scattering and reflecting characteristics of electromagnetic waves, FMCW obtains the reflected signals ${s}_{R}\left(t\right)$, then mixed them with transmit signal ${s}_{T}\left(t\right)$ to generate the intermediate frequency (IF) signal ${s}_{IF}\left(t\right)$. Through the processing and analysis of ${s}_{IF}\left(t\right)$, the speed, distance, angle and other information of the target object can be obtained, then the motion data of the target object is calculated to complete the perception and recognition of the target action state. Xia et al. designed a multi-channel convolutional neural network for multi-position gesture recognition using millimeter wave radar point cloud^11^. Shen et al. proposed a 3D CNN based dual-channel fusion network for feature extraction, developed a learnable relation module with neural networks as a non-linear classifier to measure the similarity between the samples of different hand gestures, and substantially enhanced the recognition accuracy^58^. Song et al. proposed a micro-motion gesture recognition network based on a Convolutional Block Attention Module (CBAM) to extract gesture features. The gesture recognition network of DenseNet and CBAM is used to recognize 12 types of micro motion gestures^59^.

### Lightweight model

Model combination can complement each other's shortcomings while leveraging the advantages of individual models, and is widely used in radar target classification and recognition research. However, these fusion models have a large volume and high resource requirements, so lightweight fusion models have more advantages. There are a large number of lightweight models studied for radar rigid target recognition, Qian et al. proposed a lightweight depth-wise separable fusion CNN (DSFCNN) for ballistic target HRRP recognition. The DSFCNN reduces the computational complexity of vanilla CNNs while improving recognition accuracy^60^. As well, they proposed a lightweight network called group-fusion 1DCNN (GFAC-1DCNN), and introduced a linear fusion layer to combine the output features of G-Convs, thereby improving recognition accuracy^61^. Xiong et al. proposed a lightweight model for ship detection and recognition in complex-scene SAR images. Experimental results have shown that this method has achieved good results^62^. The use of radar to recognize non rigid human body movements and gestures, due to the complexity of radar signals and the diversity of features, lightweight models can produce better recognition results for human body movements. Mainak et al. designed a lightweight DCNN model DIAT-RadHARNet with a total of 55 layers for human suspicious activity classification^63^. Zhu et al. proposed an extremely efficient convolutional neural network (CNN) architecture named Mobile-RadarNet, which is specially designed for human activity classification based on micro-Doppler signatures^64^. A lightweight PointNet-based classifier is custom-designed to recognize and classify arm-gestures point cloud images by Xie et al.^65^, Salami et al. have developed a Message Passing Neural Network (MPNN) graph convolution approach for millimeter wave radar point clouds^66^.

However, the above researches of gesture recognition encountered great difficulties, such as poor experience caused by wearable, privacy disclosure and limitations of lighting environment caused by camera, low perception accuracy and poor anti-interference ability of Wi-Fi and acoustic wave. Due to the significant advantages of FMCW in addressing the aforementioned issues, FMCW radar is used in this study to improve the adaptability and generalization ability of methods.

## System design

### System overview

This study proposes a gesture recognition method based on millimeter wave radar in complex scenes, which consists of data collection, data processing and feature extraction, and gesture recognition. Before data collected, 14 gestures are designed, which contained BFB, CWF, DUD, FBF, FP, GRASP, LRL, OK, RLR, SF, THUP, DUD, Z and circle. In order to verify and analyze the various signal features caused by the difference of range, velocity and direction of gesture actions, The design of gestures takes into account the motion range, direction, speed and other factors. FMCW radar is used to collect data. The IF signal is generated by mixing the transmitted and reflected signals, sampled as discrete data, and stored on disks. The original data sequence is reconstructed into a three-dimensional matrix according to the number of receiving antennas, frames, and chirps in the data processing and feature extraction step. The second-order feedback filter Moving Target Indication (MTI) processes the feature information further, filtering the clutter and interference. Fast Fourier transform (FFT) works along with the different dimensions of the three-dimensional matrix, and different features are obtained.

**Figure 1:**
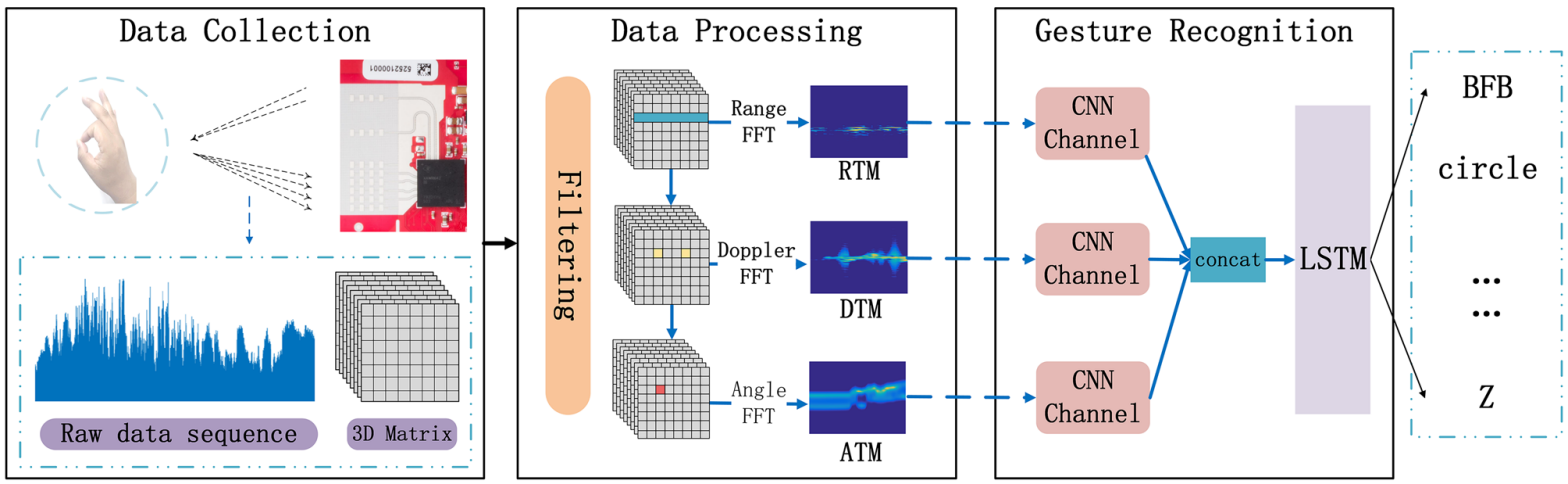
Workflow of gesture recognition system.

In gesture recognition stage, a Multi-CNN-LSTN model is proposed. The convolution features of different gesture feature that each CNN extracts are fused; LSTM completes the classification and recognition of the fused features. The workflow of the system is shown in Fig. 1.

### FMCW signal modulation

**Figure 2:**
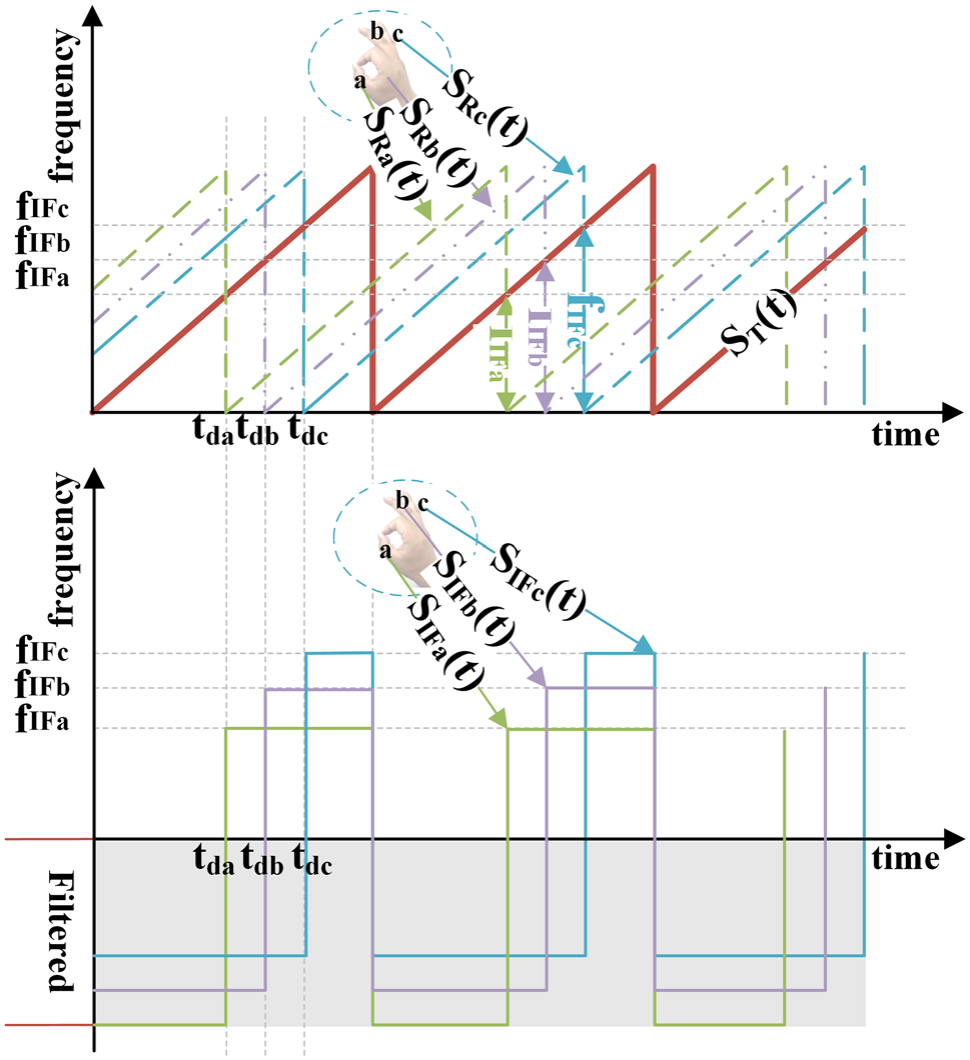
FMCW signal modulation.

In this study, 77 GHz FMCW radar is used to capture the gesture signal, and the FMCW signal modulation is shown in Fig. 2.

Taking three different parts a, b and c of a gesture as examples, the acquisition process of the gesture signal is described as follows: as the signal ${s}_{T}\left(t\right)$ is transmitted, a, b, c reflected signals as ${s}_{Ra}\left(t\right)$, ${s}_{Rb}\left(t\right)$, ${s}_{Rc}\left(t\right)$ after the delay ${t}_{d}=\frac{2\times R}{c}$, which has the time difference ${t}_{da}$, ${t}_{db}$, ${t}_{dc}$ with the ${s}_{T}\left(t\right)$ respectively. So, the radar will get three IF signals ${s}_{IFa}\left(t\right), {s}_{IFb}\left(t\right), {s}_{IFc}\left(t\right)$ with frequency of ${f}_{IFa}$, ${f}_{IFb}$, ${f}_{IFc}$ respectively. The IF signals ${s}_{IFa}\left(t\right)$, ${s}_{IFb}\left(t\right)$, ${s}_{IFc}\left(t\right)$ are then sampled by DCA at a frequency of 5 MHz, the discrete data are stored in the sampling order.

### Gesture signals and features extraction

**Figure 3:**
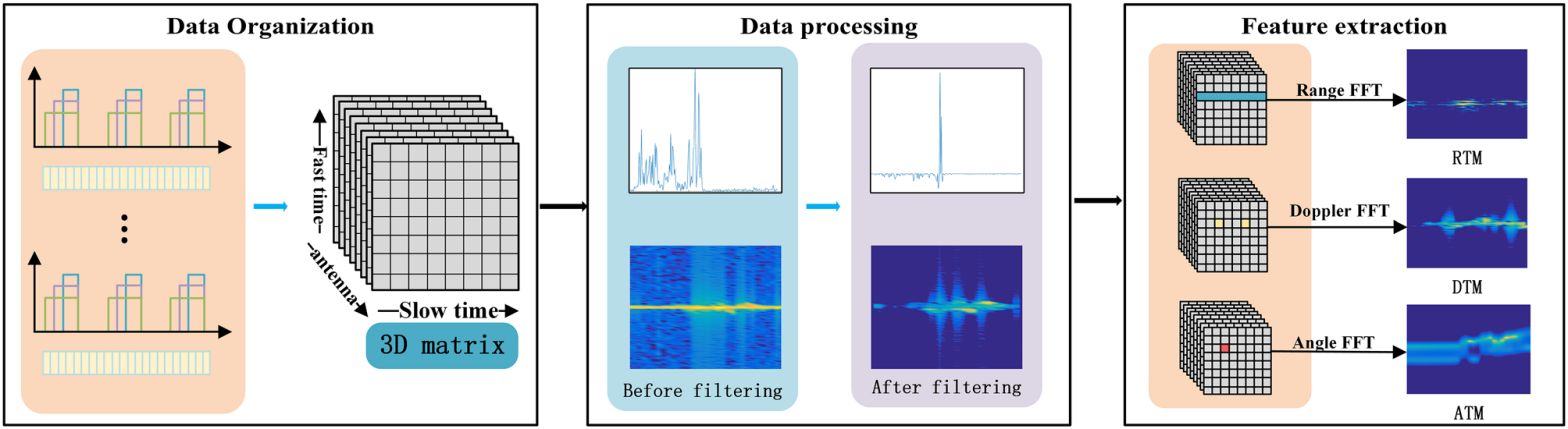
Workflow of signal processing.

In order to extract gesture information from the obtained gesture data, the ADC data is reorganized and stored as certain logical relationship; the corresponding processing methods are adopted according to different signal units to extract gesture features. The workflow of signal processing is shown in Fig. 3, which is mainly completed in three stages. The first stage is data organization, which provide data with logical relationship. The second stage is data processing, which mainly completes data reorganization, clutter filtering and time–frequency characteristics analysis and processing. The third stage is gesture feature extraction, which produce the RTM, DTM and ATM features that can effectively represent a gesture.

### Gesture signals processing

(a) Data organization and clutter filtering

The original IF signals of gestures IQ-modulated are sampled and stored as a binary sequence. First, it is represented as complex signals according to the storage rule, then the complex signals are reorganized into a three-dimensional matrix with size of {NRXs, Nframes, NChirps}, according to the logical relationship of the data, where NRXs = 4, Nframes = 32, NChirps = 128 represent the number of receiving antennas, frames per gesture, and chirps per frame, respectively. For the data from each receiving antenna, each column is a chirp means fast time, each 128 chirps consist a frame, each gesture has 32 frames, 4096 chirps. The row direction is slow time. Coherent pulse accumulation is used to improve the signal strength and enhance SNR. Then Butterworth filter and MTI algorithm are used to eliminate static clutter which is resulted by the environment around the gesture, such as walls, tables, chairs, and other static objects. The velocity and distance of static clutter signal are fixed. According to the range Eq. (1) from gesture to radar:

1 $$R=\frac{c{f}_{IF}}{2k}$$

where c is the speed of light constant, ${f}_{IF}$ is the frequency of the IF signal, and $k$ is the FM slope, which takes the value of 2401.44 $MHz/\mu s$ in the paper.

Known ${f}_{IF}=\frac{2kR}{c}$, so the frequency of the static clutters IF signals remains constant in the whole gesture signal, and the gesture distance $R\in \{r|0.1m<r<0.8m\}$ in this paper, so the IF frequency of the gesture ${f}_{IF\_gesture}\in \{{f}_{IFg}|26.7Hz<{f}_{IFg}<213.5Hz\}$, clutter frequency ${f}_{IF\_clutter}\in \{({f}_{IFc}|<26.7Hz)\cup {(f}_{IFc}>213.5Hz)\}$. Therefore, Butterworth passband filter is designed to filter clutter.

After Butterworth passband filtering, static clutters are filtered. But there are still reflected signals from motion object in the spectrum, such as arms, bodies and objects in multipath distance, which also dramatically impacts the signal. Therefore, a filtering algorithm ValScale-VAR (variance-based numerical scaling algorithm) is proposed to further eliminate clutter and suppress noise. The ValScale-VAR filtering algorithm first decomposes the FFT matrix into the same frequency units composed of different pulses and the same sampling point, and finds the sequence corresponding to the maximum variance. Then it calculates the variance in the same fast time direction according to the found maximum variance sequence, finds the maximum variance, and numerically scales the found corresponding rows and columns, so as to filter out the clutter.

(b) Time frequency characteristics of gesture signals

The fundamental difference between a gesture and others lies in the different states of each part of the hand in the spatio-temporal sequence, and the most significant of these spatio-temporal states are distance, speed, angle, time and their relationship. Through radar signal processing and using signal parameters to determine the distance, speed, angle and space–time relationship of hand gesture, the representation characteristics of hand gesture in radar signal are formed. According to Eq. (1), the range is directly related to the frequency of the IF signal, and the IF signal is converted from the time domain to the frequency domain to obtain the frequency characteristics of the IF signal, thus obtaining the distance characteristics. The speed of the target gesture is:

2 $$\upsilon =\frac{\Delta \phi \lambda }{4\pi {T}_{C}}$$

It follows that the velocity is related to the phase difference $\Delta \phi$ of the neighboring chirp. The estimation of ${f}_{IF}$ and $\Delta \phi$ requires the conversion of the radar signal from the time domain ${s}_{IF}\left(t\right)$ to the frequency domain ${s}_{IF}\left(f\right)$ using the FFT:

3 $${s}_{IF}\left(f\right)=\sum_{t=0}^{N}{s}_{IF}\left(t\right)\text{exp}(-\frac{j2\pi ft}{N})$$

Different targets with the same distance generated IF signals with the same frequency regardless of their speeds. The fast time FFT obtains a single peak signal to represent the merged signals of these target objects with different speeds of motion from the same distance. These signals can only determine the distance of the target, but not the velocity. The different velocity of the gestures will be represented in the adjacent sampling units of different chirps. Therefore, the slow time FFT is performed to obtain the velocity. After fast time FFT and slow time FFT implemented on the IF signals, variety distances and velocities are obtained. Each receiving antennas of the radar received same echo signals with different distances. FFT is performed along the antenna sequence to obtain the angle information. After processing the echo signals, the data including distance, speed and angle are obtained.

Take frame sequence as time information, a feature image is created to represent the motion state of the hand. According to the system design of this paper, the distance-time, Doppler-time, and angle-time features of various designed gestures are extracted to represent a gesture.

#### Gesture features extraction

RTM, DTM, ATM are extracted from signals to represent a gesture.

(a) RTM features

**Figure 4:**
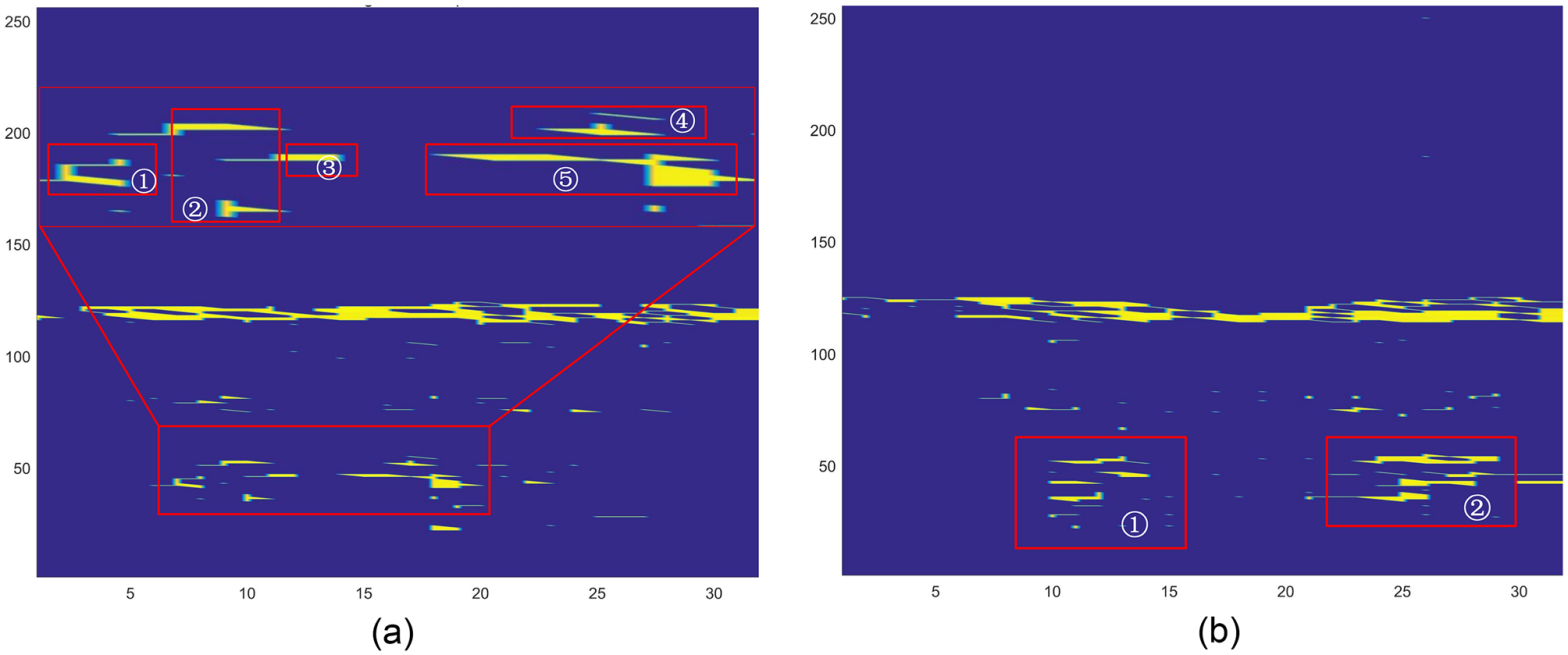
RTM features analysis.

The RTM responds to the transformation of the relative distance between the target and the radar over time. The frequency amplitude relationship information is obtained through fast-time FFT for each frame, and the frequency amplitude information of all frames is combined according to the frame sequence to obtain the RTM. Figure 4 analyzes the information contained in the gesture RTM feature in detail. Figure 4a is the RTM feature picture of the SF (snap finger) gesture. Combined with the specific action of the SF gesture, the palm is downward at an angle of 45°–60° with the horizontal desktop, the fingers are naturally extended and slightly bent, and the fingers are 45°–60° with the plane of radar equipment. When making snap finger movement, the little thumb, ring finger, and middle finger are bent to the palm in turn, the thumb is closed to the middle finger, the index finger is turned naturally with the palm, the finger is pulled back, and the hand is slightly turned locally. After the thumb and middle finger are in close contact, the whole gesture movement pauses for a short time (< 1 s), and then the thumb and middle finger move in the opposite direction, rubbing each other to make a sound. The hand continues to turn until the palm is entirely up. Therefore, the hand positions of RTM feature marks in the figure are as follows: ① it indicates that the wrist and back of the hand rotate with the arm as the axis at the beginning of the action, and the distance does not change significantly; ② indicates that the slightly separated index finger, middle finger, ring finger and little thumb move backward with the rotation of the wrist; ③ indicates that after rubbing with the thumb, the middle finger continues to move backward until it stops; ④ continue to move the thumb forward until it stops; ⑤ indicates the continuous state after the completion of the whole gesture. Similarly, Fig. 4b is the RTM feature map of the grasping gesture. ① represents the distance feature of four fingers except for the thumb in the process of gradually grasping, ② represents the movement process of the thumb.

(b) DTM features

**Figure 5:**
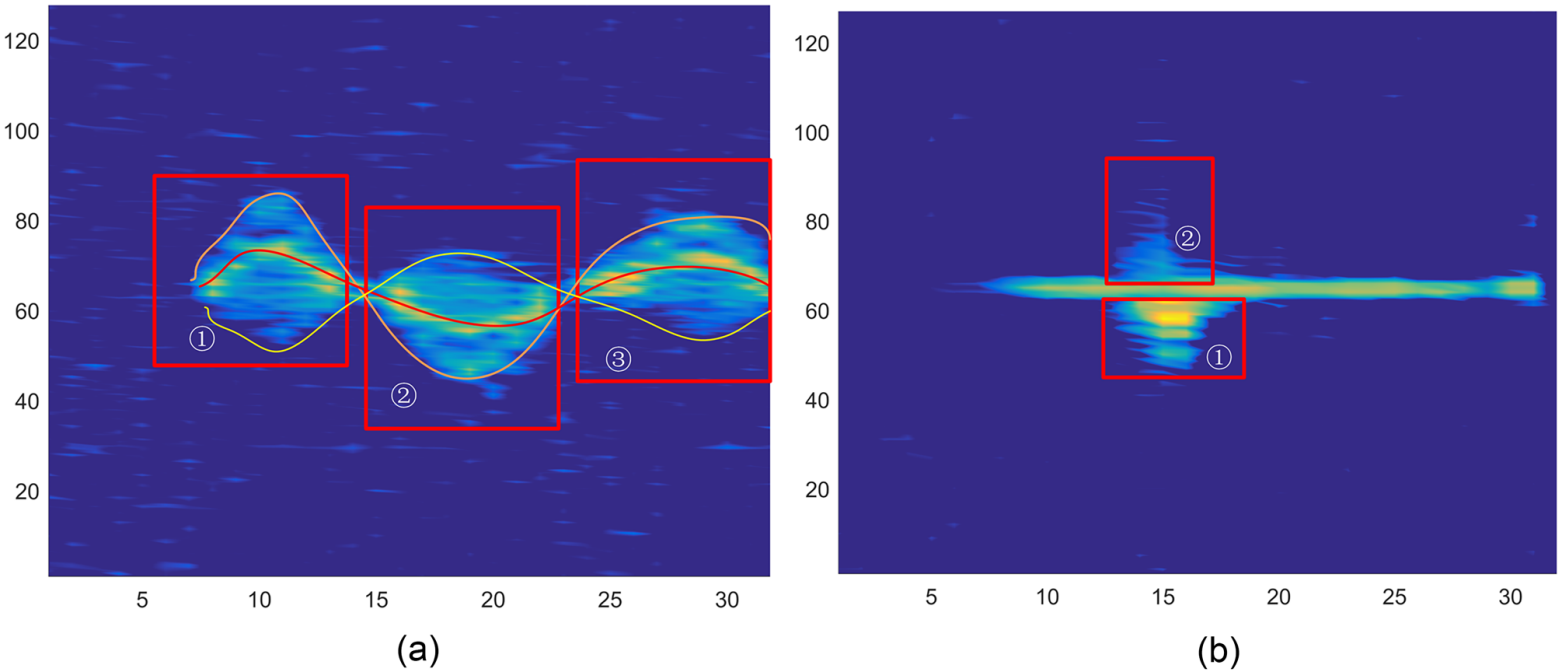
DTM features analysis.

The fast time FFT obtains the distance information in a frame, based on which the slow time FFT obtains the RDM. The Doppler feature position in RDM is determined according to the feature position of RTM, and the Doppler time feature is accumulated frame by frame. In Fig. 5, RDM features of two kinds of gestures are shown. Figure 5a is a BFB (back-forward-back) gesture. In the figure, red, yellow, and orange lines represent Doppler time features of different arm parts. ① During the period, the gesture starts to move from the static state close to the radar, reaches the fastest speed, and stops at the position far away from the radar. ② During the period, the gesture starts to move from the static state far away from the radar and stops at the position close to the radar after passing the maximum speed. The same is true in stage ③. The stages ①, ②, and ③ show that different parts have different Doppler velocities, and the positive and negative values of velocities in different directions are different. Figure 5b is the DTM information of the FP (flipped palm) gesture. FP gesture is a relatively simple gesture from which the Doppler information of gesture can be seen intuitively. FP gesture is to naturally extend the palm, palm up, and then turn to palm down. It can be seen from part ① in the figure that in the process of turning the palm, the turning of the palm takes the middle finger line as the axis. The thumb and index finger move away from the radar direction, as shown in part ① in the figure, while the ring finger and little thumb move towards the radar, as shown in part ② in the figure. So, there is different Doppler information for the same period. The information characteristics of action and distance can be seen in the figure.

(c) ATM features

**Figure 6:**
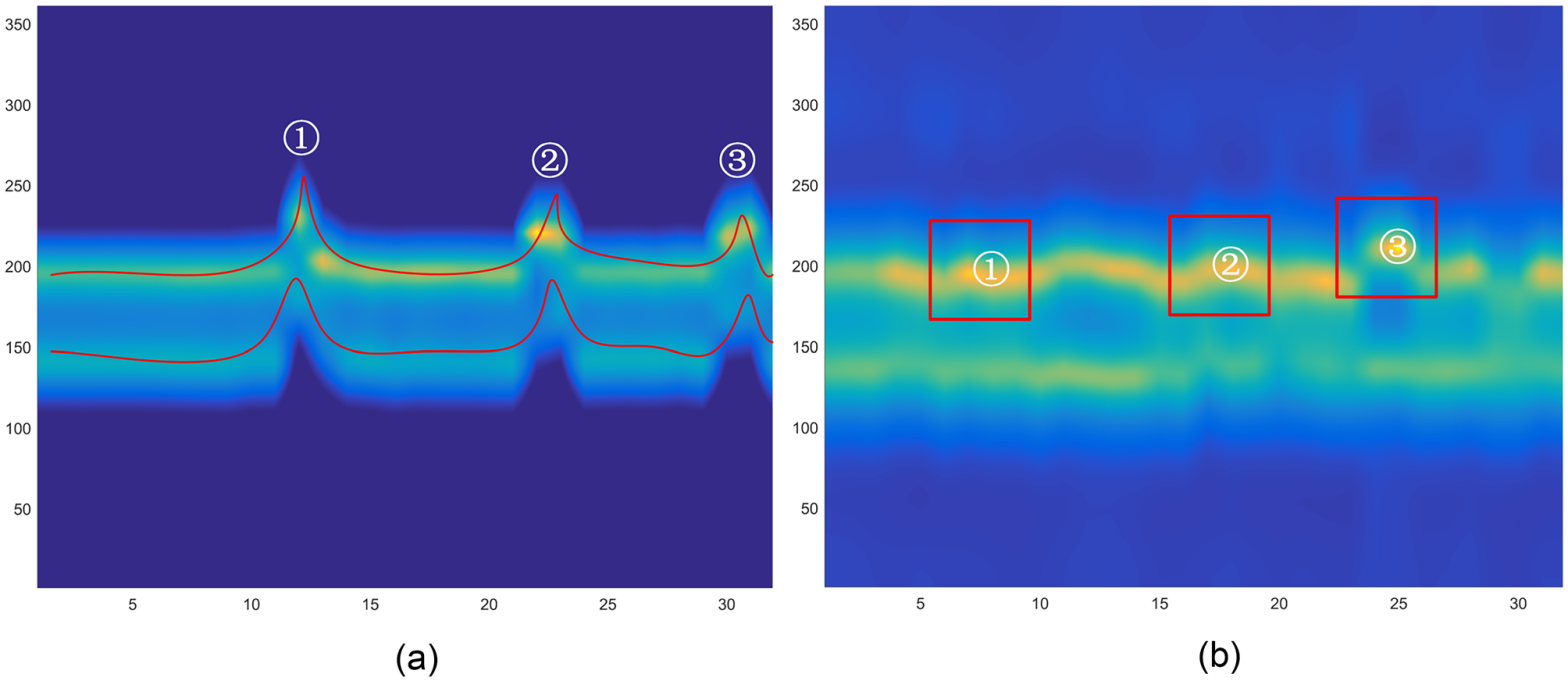
ATM features analysis.

According to the propagation distance difference of signals received by different antennas, the angle information of the incident signal can be determined according to the distance between antennas:

4 $$\theta ={\text{sin}}^{-1}\frac{\Delta \phi \lambda }{2\pi l}$$

where $\Delta \phi$ Is the phase difference, and l is the distance between two antennas. Figure 6 shows the time angle characteristics of UDU and CWF gestures. In Fig. 6a, there are three angle peaks, respectively showing the angle change state during the up and down movement of UDU hand. As the hand is lifted upwards, the angle gradually increases. ① represent the first maximum angle when the palm reaches the top. Then palm drops to its lowest point, the angle get the second maximum again, as is shown by ②. The upper and lower curves in the Fig. 6a represent the different parts of physical. The variety motion range of each part generated variety angles. The above curve depicts the angle variation tendency of the fingertips, while the following curve describes the wrist. Figure 6b clearly shows the change of angle information of CWF gesture with time, and clearly sees the peak value and change of angle at different times and positions during the duration of gesture.

### Neural network models design

A gesture is represented by RTM, DTM, and RDM features obtained by radar IF signal processing.

These feature images fully express the distance, speed, angle, and time of gestures, all of which contain important gesture features. CNN can perfectly extract key features from feature images and reduce computational complexity. By designing a three channels CNN for extracting convolutional features from RTM, DTM, and ATM images, respectively. Dynamic gestures are sequences of spatiotemporal states of the arm, and temporal information and the long short-term dependencies of gesture states are important information for dynamic gestures, which play an important role in gesture differentiation. The LSTM model can perfectly record the long short-term dependencies of data, so LSTM is introduced into the design model.

**Figure 7:**
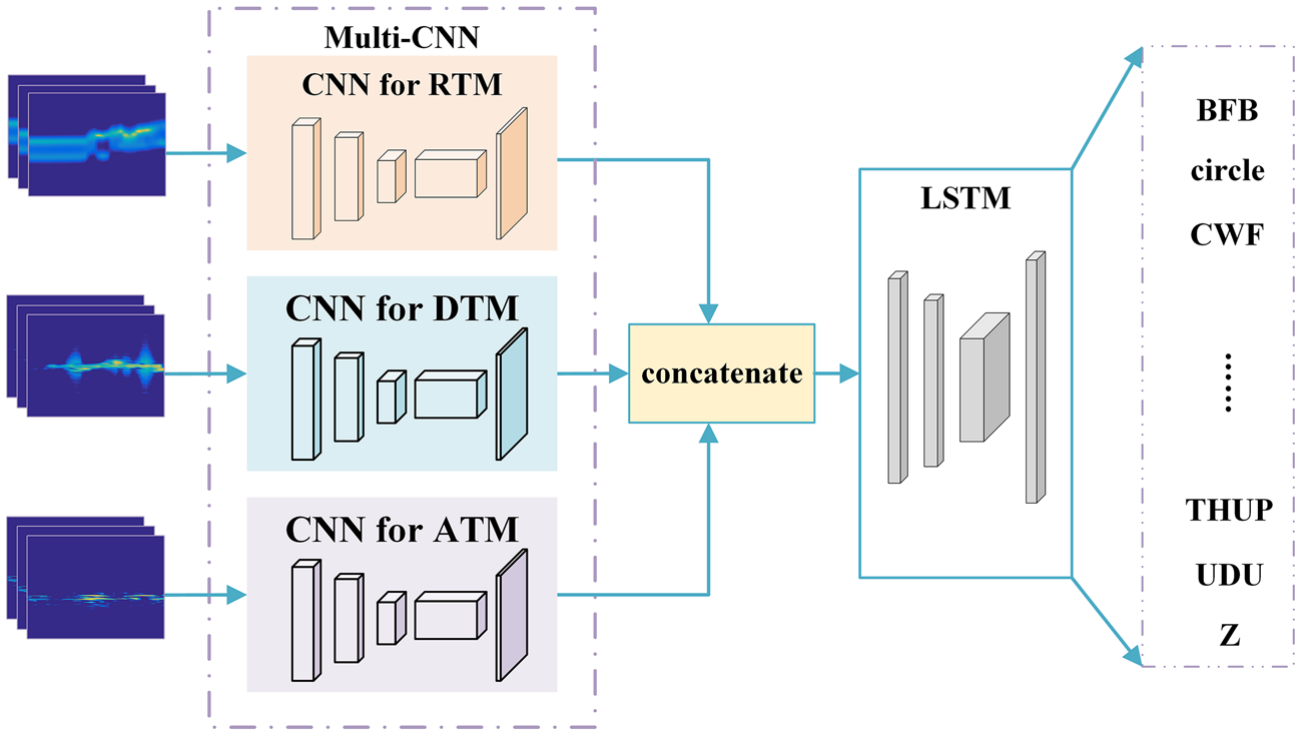
Multi-CNN-LSTM model design.

In this study, the Multi-CNN-LSTM neural network model is designed to recognize hand gestures respectively; RTM, RDM, and DTM features are fed into three CNN channels, and each channel extracts the convolutional features of the different feature images, the convolutional features extracted from the three channels are fused, and fed into the LSTM. Finally, the classification and recognition are completed by Softmax. The Multi-CNN-LSTM model is shown in Fig. 7.

Multi-CNN proposed in this paper consists of three CNN channels with the same structure with an input size of 28 * 28 * 3 and an output size of 28 * 28 * 16. The convolutional layer completes the down sampling and reduces the input size. The Conv2D convolutional kernel size is 5 * 5 * 16. ReLu is used as the activation function to improve the fitting ability of its nonlinear factors and enhance the expression ability of the convolutional layer for complex features. To prevent the excessive training parameters, a Maxpool2D is added with a pooling size of 2 * 2 and an output size of 14 * 14 * 16. After the convolution and pooling operations, the convolutional features are obtained. To prevent overfitting issues, set dropout = 0.4 for the model.

An input RGB image with size of 28 * 28 * 3 is decomposed into R, G, and B three images with size of 28 * 28. convolution operation is performed on each image to obtain the convolutional features of the input data. The convolution operation for each single channel graph is:

5 $$F_{output} \left( {x,y} \right) = F_{input} \left( {x,y} \right) \otimes H_{core} \left( {x,y} \right) = \mathop \sum \limits_{i = 0}^{k - 1} \mathop \sum \limits_{j = 0}^{l - 1} F_{input} \left( {x + i,y + j} \right) \times H_{core} \left( {x,y} \right)$$

**Figure 8:**
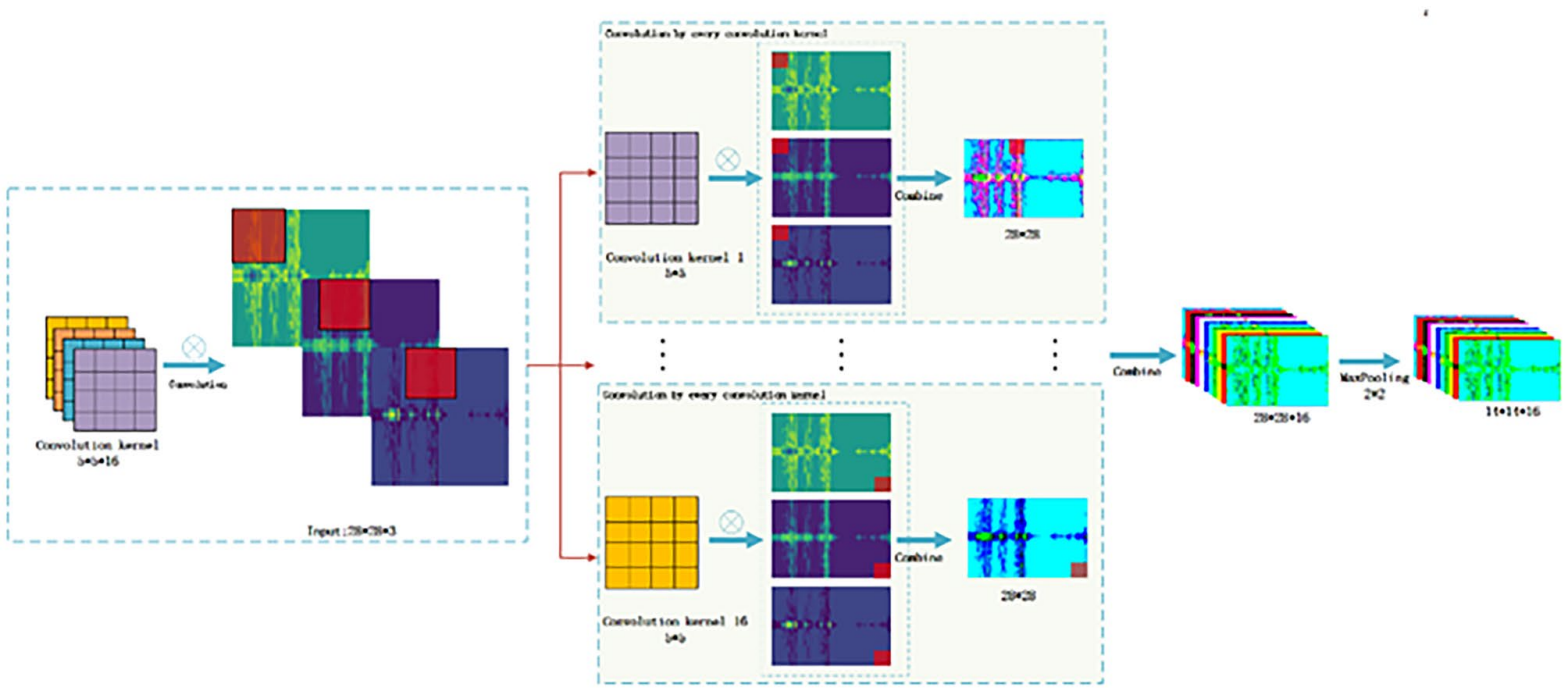
CNN feature extraction process.

${F}_{output}\left(x,y\right)$ is the result of convolution operation, ${F}_{input}\left(x,y\right)$ is the input data of 28 * 28 size, and ${H}_{core}\left(x,y\right)$ is the convolution kernel of $k\times l$ size, k = 5, l = 5, ⨂ is the convolution operator. The convolution operation process for each input RGB image in this paper is shown in Fig. 8.

After convolution and pooling operations, the convolutional features of three CNN channels are fused as an input to the LSTM model. LSTM is a classic recursive neural network model that can express the dependency relationship of input sequences. The input with a set time step and the output of the previous time step are fed to the LSTM unit, and the generated result is input to the next unit. The following equation represents the update of LSTM units:

6 $$\left\{ {\begin{array}{*{20}l} {{\text{C}}_{t} = F_{t} \odot {\text{C}}_{{\left( {t - 1} \right)}} + I_{t} \odot {\tilde{\text{C}}}_{t} } \hfill \\ {{\text{I}}_{t} = sigmoid\left( {H_{{\left( {t - 1} \right)}} w_{hi} + X_{t} w_{xi} + b_{i} } \right)} \hfill \\ {{\tilde{\text{C}}}_{t} = \tanh \left( {H_{{\left( {t - 1} \right)}} w_{hc} + X_{t} w_{xc} + b_{c} } \right)} \hfill \\ {F_{t} = sigmoid\left( {H_{{\left( {t - 1} \right)}} w_{hf} + X_{t} w_{xf} + b_{f} } \right)} \hfill \\ {{\text{O}}_{t} = sigmoid\left( {H_{{\left( {t - 1} \right)}} w_{ho} + X_{t} w_{xo} + b_{o} } \right)} \hfill \\ {{\text{H}}_{t} = {\text{O}}_{t} \odot \tan h\left( {{\text{C}}_{t} } \right)} \hfill \\ \end{array} } \right.$$

LSTM consists of three gates and memory cells, with the input gate containing ${\text{I}}_{t}$ and ${\widetilde{\text{C}}}_{t}$. Forgotten Gate contains ${F}_{t}$. The output gate contains ${\text{O}}_{t}$ and ${\text{H}}_{t}$. ${\text{C}}_{t}$ is the cellular state, ${\widetilde{\text{C}}}_{t}$ is a candidate memory cell. ${\text{H}}_{t-1}$ is a hidden state, representing short-term memory; ${\text{C}}_{t-1}$ is the cell state, representing long-term memory, ${X}_{t}$ is the input, $w$ is the weight, $b$ is the offset, the information needs to be recorded of ${X}_{t}$ is provided to the cell state ${\text{C}}_{t}$ by the hidden state ${\text{H}}_{t-1}$. “⨀” is the multiplication of the pairwise elements of two matrices, and “+” is the addition of the pairwise elements of two matrices. ${F}_{t}$ determines the number of forgotten internal states, and the output gate regulates the impact of internal states on the system.

## Experiment and result analysis

This section evaluates the overall performance of gesture recognition methods through experiments. We introduced the experimental environment and data collection detailed. The recognition effectiveness of feature fusion is verified by compared the results of single-features and fused-features. The performance of Multi-CNN-LSTM is analyzed. Firstly, we compared the accuracy and resource requirements with other various models, then we analyzed the performance under imbalanced samples and the complexity of model. The model’s robustness of scenario variety, personnel variety, velocity variety and location variety.

### Experimental settings

Hardware configuration: AWR1642 radar is applied in the experiment, which supports operating frequencies of 77–81 GHz with a maximum bandwidth of 4 GHz. This device supports two transmitting antennas and four receiving antennas. The data acquisition uses TI's DCA1000 high-speed data acquisition card, which obtains the intermediate frequency signal output by the AWR1642 radar device. The ADC sampling frequency set in the experiment is 5 MHz, and the sampling data is transmitted to a PC through a gigabit Ethernet. The experimental PC operating system is Windows 10-64bit, AMD Ryzen 5 CPU@2.00 GHz 8 GB memory, 2 GB graphics card. The main configuration of AWR1642 and ACD1000 equipment in the experiment is shown in Table 1, according to the radar distance resolution equation:

7 $${R}_{Res}=\frac{c}{2B}$$

we can calculate that the distance resolution is 6.25 cm, according to the radar velocity resolution equation:

8 $${\upsilon }_{res}=\frac{\lambda }{2{T}_{f}}$$

we can calculate that the speed resolution is 4.87 cm/s.

**Table 1:** Parameters configuration of radar equipment.

Radar parameter name	Value
FM bandwidth	2.4 GHz
antenna	2Tx, 4Rx
FM periodicity	60 µs
Frame periodicity	40 ms
Number of chirp loops in a frame	128
Sampling rate	5 MHz
ADC samples (Number of sampling)	256
Distance resolution	6.25 cm
Velocity resolution	4.87 cm/s

Software implementation: The radar software platform used for data collection is mmWave Studio, and data signal processing and feature extraction are completed through Matlab2018. The fusion model is developed using Python on the PyCharm development platform, and the TensorFlow framework is used. The hyperparameter settings for model training are as follows: using an Adam optimizer with a learning rate of 0.001, batch size of 32, and number of epochs of 40.

### Data collection

**Figure 9:**
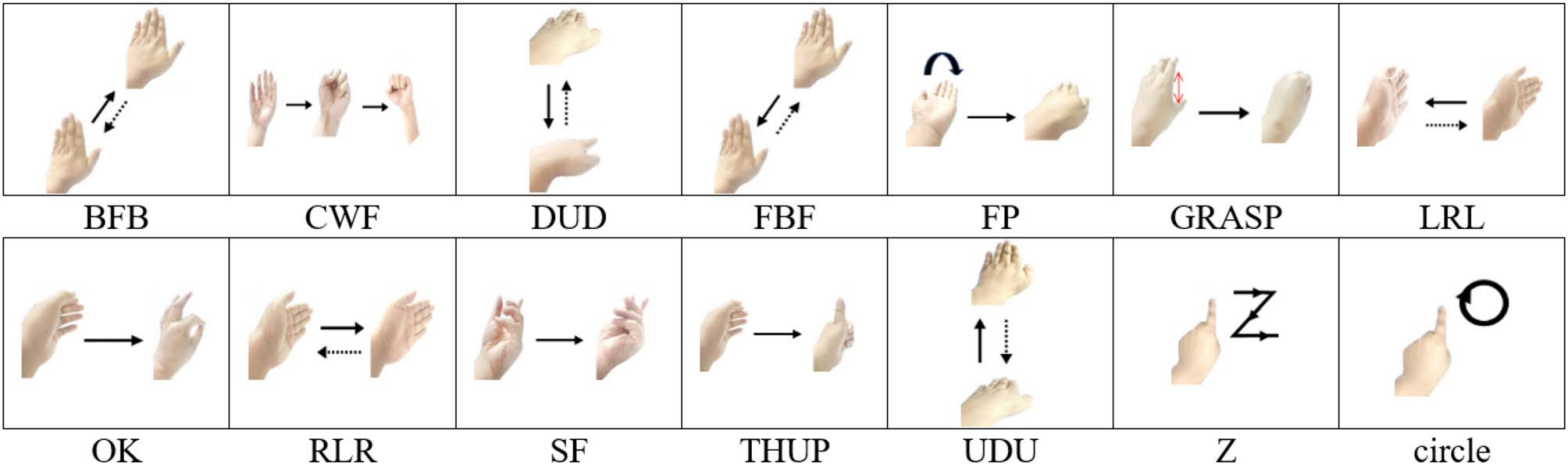
Experimental gestures design.

Based on factors such as the amplitude, speed, and direction of common gesture movements in daily life, and to comprehensively verify the perception and recognition effect of radar equipment on different factors,14 gestures were designed as are shown in Fig. 9, including different motion amplitudes, directions, angles, and speeds. The vertical thumb and snap finger movements are mainly micro finger movements, while push–pull, lift-press are mainly more significant arm movements. Pushing and pulling are movements parallel to the radar surface, swinging left and right are movements perpendicular to the radar surface. The fist-waving celebration gesture combines hand and arm movements, with richer Doppler spectral characteristics. The experimental gestures are shown in Fig. 9.

**Figure 10:**
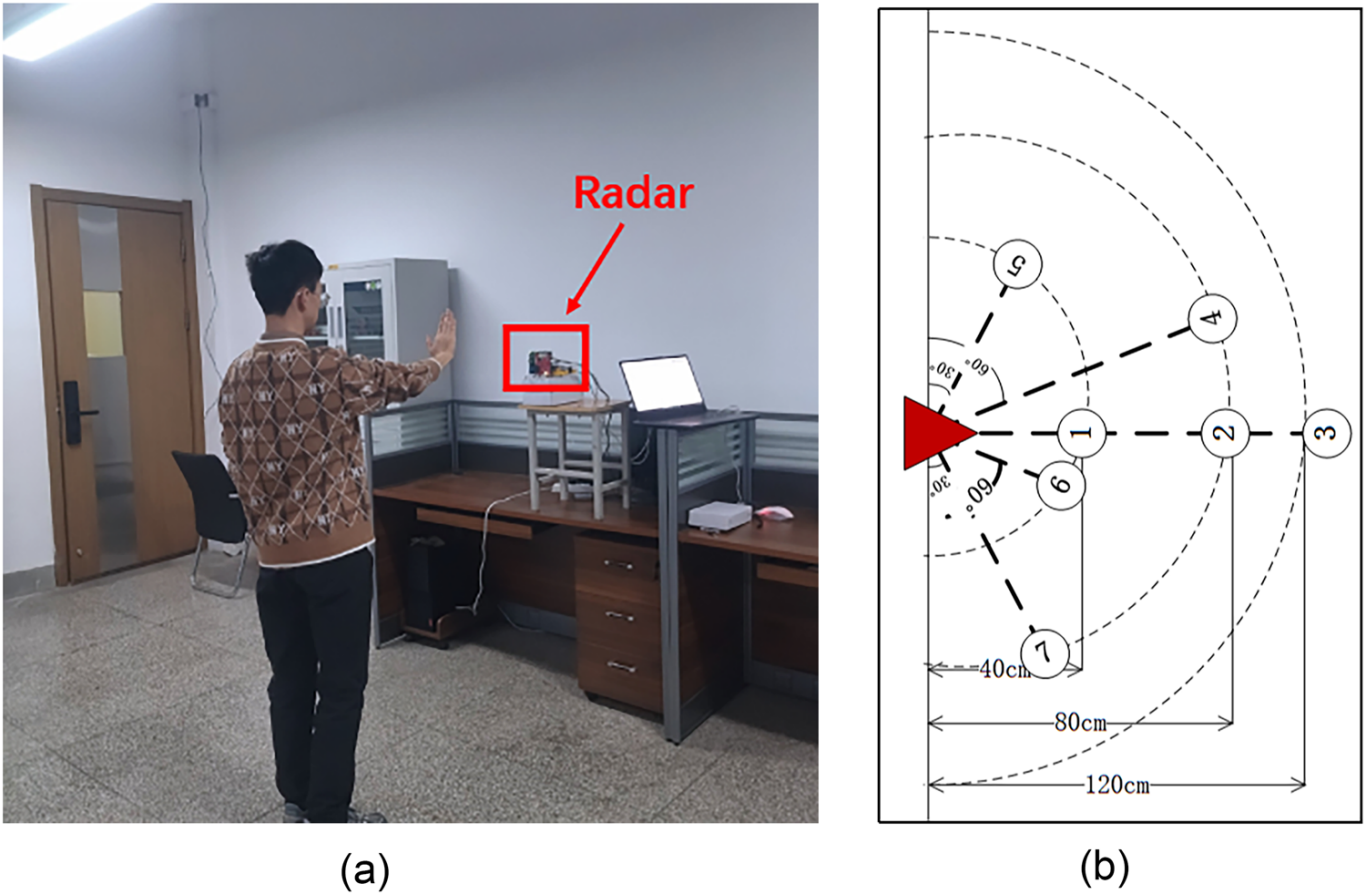
Data collection environment. (The person in (**a**) is author of this study).

The experimental environment variety lead to differences in data collection. In order to ensure the model's adaptability to complex environments, experimental data collection is completed from four scenarios: laboratory, corridor, dormitory, and outdoor. The environment of data collection is shown in Fig. 10. In Fig. 10a, the author has participated in the data collection. Three distances of 40 cm, 80 cm, and 120 cm and three angles of 30°, 60°, and 90° designed. A total of seven points were selected to collected data. Three people participated in gesture data collection; the physical information of participator is shown in Table 2. Each gesture collected 60 times by each participator in each scenario, a total of 14 gestures with 10,080 samples. The collected data is randomly divided into training set and validation set in a 6:4 ratio.

**Table 2:** Physical information of experimental personnel.

Data collection participators	A	B	C
Height (cm)	178	170	173
Weight (Kg)	63	56	52
Gender	Male	Male	Female

This is a large amount of data, which is divided into 14 groups with 2520 samples in each group. The samples are presented in the form of images, and each image is provided as input to the neural network model Multi-CNN-LSTM with dimensions of 3*28*28.

### Analysis of experimental results

To verify the overall performance of the recognition method proposed in this paper, we organized from three aspects: the features verification, the performance of Multi-CNN-LSTM and the robustness of method. As RTM, DTM, and ATM features are extracted and fused, it is necessary to verify the effectiveness and correctness of features. Then six neural network models including classical neural network models VGG19, ResNet50, MobileNet, and self-designed neural network models CNN, LSTM, and Single-CNN-LSTM are employed to compare with Multi-CNN-LSTM, verified its performance. Finally, by compared the experimental results of variety scenes, personnels, locations and velocity, the robustness of method is verified.

#### The correctness and effectiveness of features

**Figure 11:**
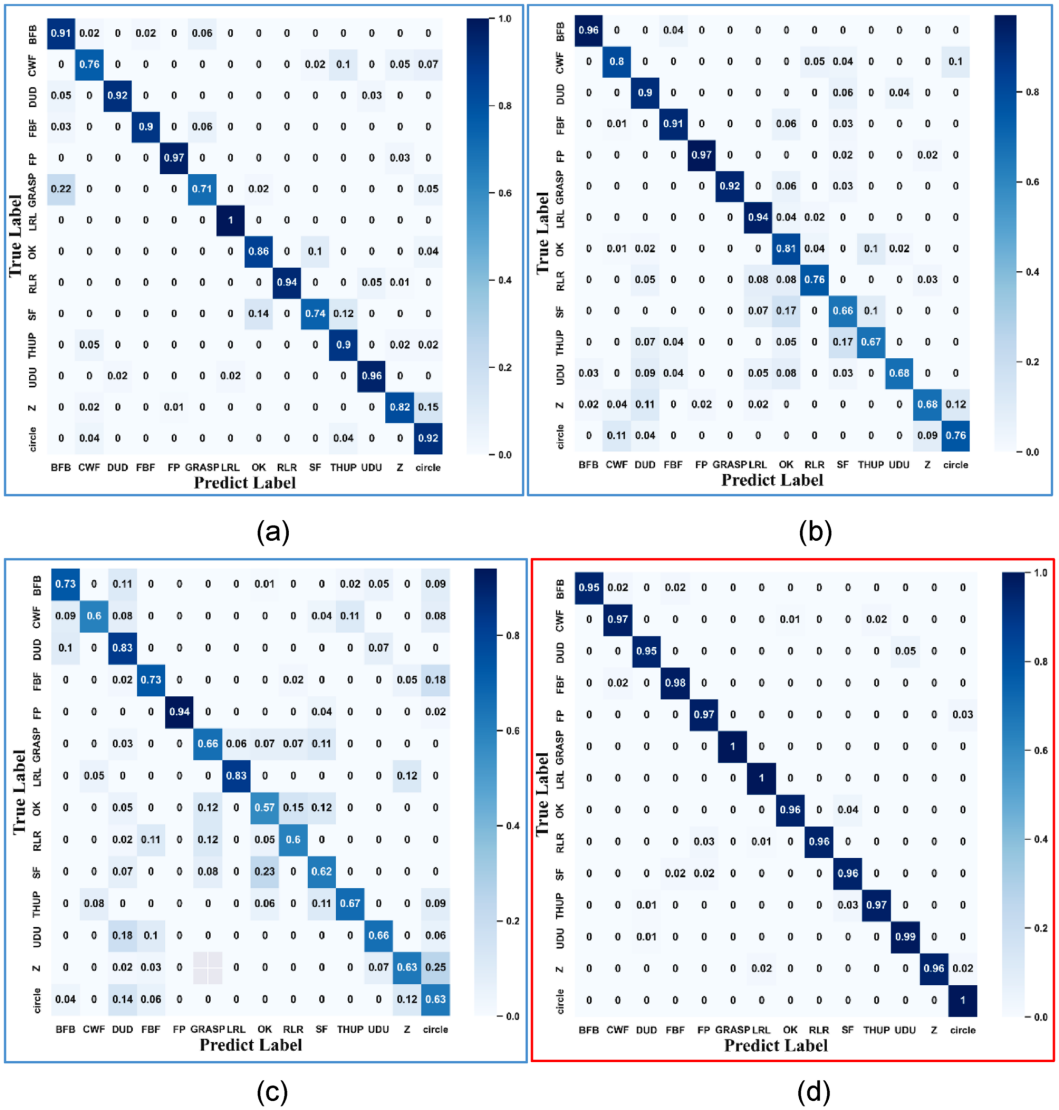
Confusion matrix of various features.

We analyzed and compared the recognition results of single-feature RTM, DTM, ATM and fused-feature, analyzed the limitations of single features, and verify the reliability and advantages of fused features. From the experimental results, it can be seen that the same gesture feature exhibits clear patterns of misidentification and confusion, which exposed the effectiveness and limitations of gesture features. RTM, DTM, and ATM are used in experiment for gesture recognition to verify the correctness and reliability of features extraction. Meanwhile, the inherent defects of each feature are exposed. Fused-features deliver results to verify the effectiveness and correctness of feature fusion. Confusion matrix of all features generated by Multi-CNN-LSTM are shown in Fig. 11. From the confusion matrix in Fig. 11, it can be seen that there is a clear pattern of confusion and misidentification of experimental gestures in the neural network model with a single feature as input.

Taking ATM as input feature, as shown in Fig. 11a, the LRL gesture has the highest recognition accuracy of 100%. The most significant statistical features of misidentification are the GRASP and Z gestures. The GRASP gesture has a 22% probability of being recognized as BFB, the Z gesture has a 15% probability of being misrecognized as circle gesture, and the SF gesture has a 14% and 12% probability of being recognized as OK and THUP gestures, respectively. The least significant statistical characteristic of misidentification rate is the CWF gesture, which has a similar probability of being misidentified as Circle, THUP, Z, and SF gestures.

Figure 11b shown the confusion situation of considering DTM as the input feature. The BFB and FP gestures have the highest recognition accuracy of 96% and 97%. The SF gesture has the most significant misidentification statistical feature with a 17% probability of being misidentified as an OK gesture. The Z gesture has an 11% and 12% probability of being recognized as a DUD and circle respectively. The least significant statistical characteristics of misidentification rate are UDU, Z, and OK gestures, which are misidentified as various other gestures, with variance of misidentification rates of 0.04%, 0.23%, and 0.05%, respectively. It can be seen that these three gestures have certain similarities in DTM features for gestures with high confusion error rates.

As is shown in Fig. 11c, when RTM is used as input feature, the FP gesture has the highest recognition accuracy of 94%. The most significant statistical features of misidentification are the SF gestures has an 23% probability of being misidentified as an OK gesture, and the Z gesture has a 25% probability of being recognized as a circle gesture. The least significant statistical characteristics of misidentification rate are the OK and GRASP gestures, which are respectively misidentified as various other gestures.

DTM represents Doppler time information, and the significant confusion pattern presented by DTM as a separate feature is due to the similarity of Doppler time characteristics between predicted and real gestures. Although different gestures have completely different gesture actions, when mapping one action to DTM features while ignoring other action information, it can lead to similarity in local Doppler time features. The action forms of the OK gesture and RLR gesture differ greatly, but the probability of recognizing OK as RLR is as high as 14%, which means that these two gestures have more Doppler time similarity.

It can be seen that when a single feature is used for gesture recognition, the confusion gestures generated by each individual feature exhibit obvious statistical patterns. The confusion results generated by a single feature used for gesture recognition conform to statistical patterns, which proves the inherent limitations of this feature. Therefore, the feature information carried by individual features is effective, but not sufficient to fully identify a gesture.

The fact that various features represent various orientations leads to inherent limitation of single feature. The fusion features complement each other's inherent defects, comprehensively expressing the distance, speed, angle, and time information of gestures, thereby improving the recognition effect of gestures. Compared with the confusion matrix of the single features in Fig. 11, the confusion matrix of the fused features in Fig. 11d has a higher accuracy in the model, eliminating the possibility of gesture confusion. The model has an accuracy of 97.28% in recognizing the fused features.

#### Performance analysis of models

There are five indicators for the performance of models: Accuracy, Precision, Sensitivity, Specificity, and Negative Predictive Value. The recognition results of each gesture can be classified into four types: True Positive (TP), False Negative (FN), False Positive (FP), and True Negative (TN). We compared accuracy of models mentioned above with Multi-CNN-LSTM, and analyzed the P-R curve and ROC to confirm the performance under imbalanced samples.

(a) Analysis of model accuracy

**Figure 12:**
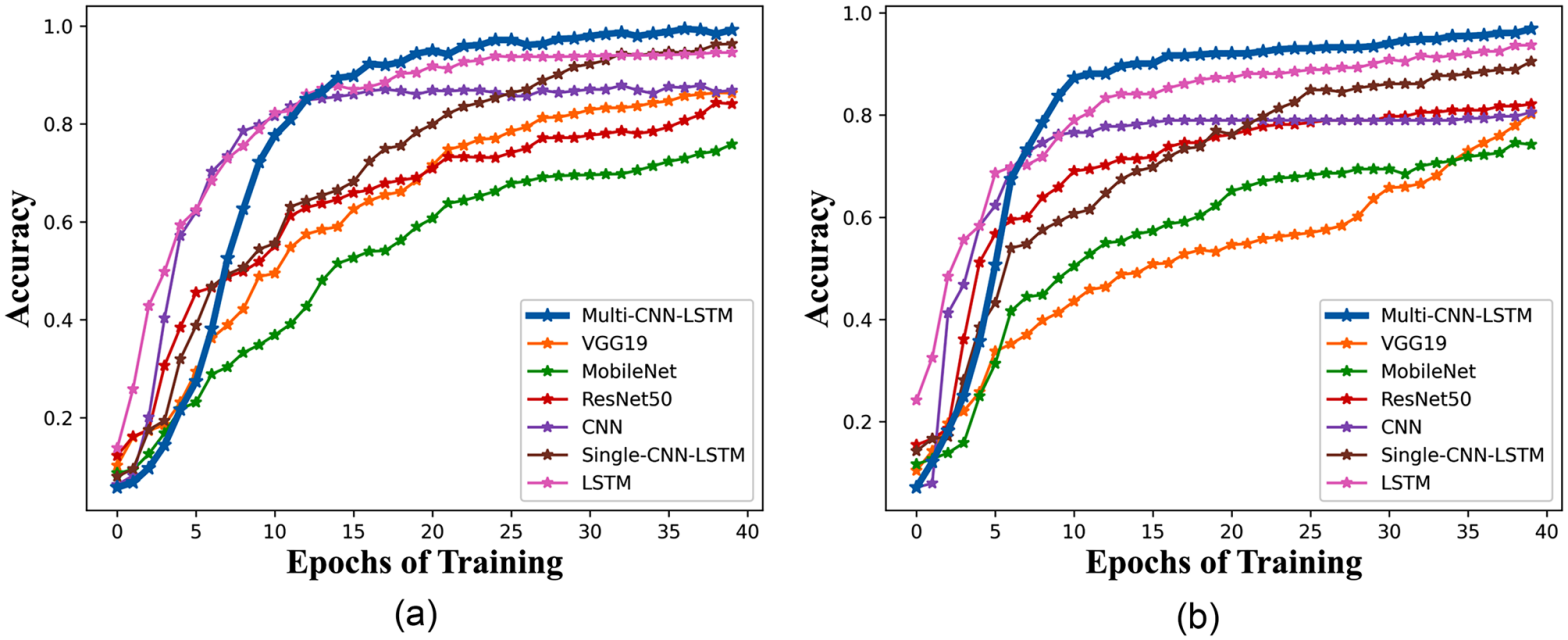
Accuracy of various models.

Accuracy refers to the proportion of correctly classified samples to the total number of samples. Different neural network models were compared with the designed Multi-CNN-LSTM, and various features were used as inputs for the experimental results. The data obtained are shown in Table 3. It intuitively reflects the accuracy and memory requirements of each model. The running memory requirements of the classic model are relatively high, with ResNet50 having a running memory requirement of 2.29 GB and VGG19 having a running memory requirement of 376.55 M. However, the self-designed model has a relatively low running memory resource requirement, with CNN having 112 M, LSTM and Single-CNN-LSTM having 28.12 M and 27.28 M, respectively. The memory requirement of the Multi-CNN-LSTM model designed in this article is 42.57 M. The accuracy of contrast models was analyzed. The highest accuracy is 94.66% of the LSTM model and the lowest being 75.85% of MobileNet. The average accuracy was 87.01%. The recognition accuracy of the Multi-CNN-LSTM model designed in this article is 97.28%, which is 2.62% higher than the highest recognition rate of 94.66% in other models. The recognition accuracy of the comparison model and the experimental model is shown in Fig. 12. Figure 12a has shown the recognition accuracy of the model in the training set, and Fig. 12b has shown the recognition accuracy of the model in the validation set. By comparison, the Multi-CNN-LSTM model designed in this article has lower memory resource requirements, higher recognition accuracy, and higher practicality. The reason why the model proposed in this article has higher recognition accuracy is mainly attributed to the following two reasons: firstly, the model better integrates gesture features, integrating speed, distance, angle, and temporal information. Secondly, CNN can better obtain local features of gesture information, while LSTM can better achieve long-term memory, complete gesture feature description of various information, and complete recognition.

**Table 3:** Results of gesture recognition using different models. Significant values are in bold.

Model name	CNN	VGG19	RESNET50	MOBILENET	LSTM	Single-CNN-LSTM	Multi-CNN-LSTM
Memory size	112.00M	376.55M	2.29G	1.05G	28.12M	27.78M	**42.57M**
Accuracy	86.83%	86.30%	84.24%	75.85%	94.66%	94.59%	97.28**%**

(b) Model complexity analysis

In Table 4, we can see a comparison of Floating Point Operations (FLOPs) and Memory Access Cost (MAC) for various models. FLOPs can intuitively reflect the time complexity of the model. From the data in the table, it can be seen that the proposed Multi-CNN-LSTM model has a FLOPs of 8.73 M, while the highest comparison model VGG19 reaches 39.037G, and the lowest FLOPs value is 0.8616 M of LSTM. In the comparison of MAC, the highest MAC model is 671 M in VGG19, while the lowest is the proposed LSTM and Single-CNN-LSTM, which are 56 K and 100 K, respectively. The MAC of the proposed experimental model Multi-CNN-LSTM is 218 K. It can be seen that the proposed model has significant advantages in both spatial complexity and spatial complexity. By compared the calculated density of these models, the highest value is 58.10 of VGG19, while the proposed model has a calculate density of 40.05. It can be seen that this model has a high calculate density.

**Table 4:** Model complexity indicator.

Model name	CNN	VGG19	RESNET50	MOBILENET	LSTM	Single-CNN-LSTM	Multi-CNN-LSTM
FLOPs	3.11G	39.04G	7.72G	598.99M	0.86M	4.45M	8.73M
MAC	700M	672M	211M	74M	56K	100K	218K
Calculate density	44.43	58.10	36.59	8.09	15.39	43.25	40.05

(c) Model overfitting analysis

**Figure 13:**
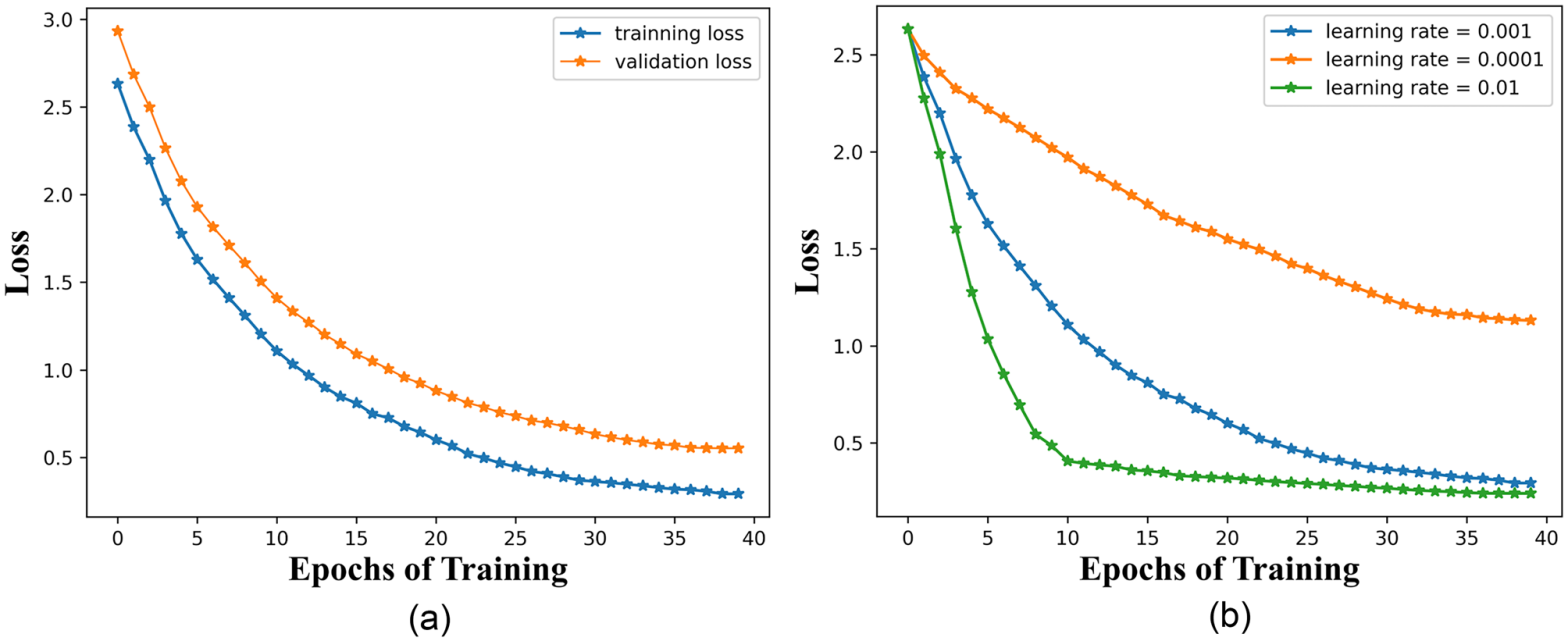
Loss of Multi-CNN-LSTM.

The overfitting problem during model training can be mainly solved from the following aspects: first, adjust the learning rate to achieve the best state, and then add dropout = 0.35 to the model to better avoid overfitting problems. The loss curve of the model is shown as Fig. 13a, in which we can see that the training results of the model are relatively good, and the measures we have taken have effectively addressed the overfitting problem. We provide the loss curves of the model with different learning rate in Fig. 13b. From the graph, it can be seen that different learning rates have different effects on loss. When we set it to 0.0001, we obtain a slowly decreasing loss curve that has not yet converged within the set epochs. However, when we set the learning rate to 0.1, there is a rapid decrease, and a learning rate of 0.001 makes the model loss decrease smoother. The model has a normal descent gradient in both the training and validation sets, and eventually tends to stabilize. At the same time, we can see from Fig. 12 that the accuracy of the model in both the training and validation sets is 97%, so the model is not overfitting.

(d) Model performance analysis under imbalanced samples

In order to provide a more comprehensive analysis of system performance, the Receiver Operating Characteristic Curve (ROC) and Precision Recall (PR) curve were presented. Accuracy is the most intuitive indicator for evaluating the classification performance of a model, but there are obvious shortcomings when the sample distribution is uneven. When the proportion of samples in different categories is very uneven, the category with a large proportion often becomes the main factor affecting accuracy. Therefore, further validation and analysis of the model recognition performance in terms of precision, sensitivity/recall, true positive (TP), and false positive (FP).

**Figure 14:**
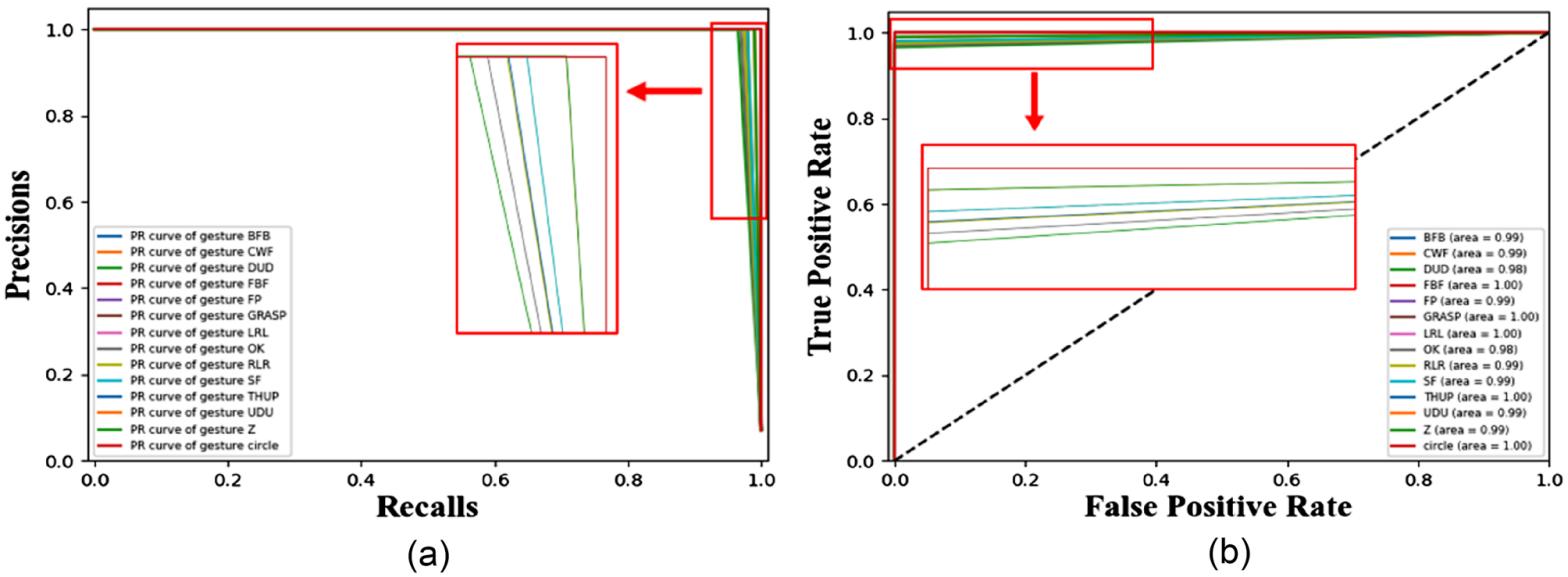
ROC curve and PR curve of Multi-CNN-LSTM.

Figure 14a shows the P-R curve of the Multi-CNN-LSTM model proposed in this paper for 14 experimental gestures. The P-R curve takes recall as the x-axis and precision as the y-axis, intuitively reflecting the relationship between the two. It shows the performance of the model under different recall and precision conditions and evaluates the classifier's recall and precision at different thresholds. From the graph, it can be seen that the recognition recall of all gestures is close to 1, while the difference in accuracy is not significant. The PR curve of the circle gesture include other gestures, which means that the recall rate is higher and the model has better recognition performance for the circle gesture. Figure 14b shows the ROC of the Multi-CNN-LSTM model proposed in this paper for 14 experimental gestures. The ROC has a FPR as the x-axis and a TPR as the y-axis. According to the meanings expressed by TPR and FPR, ROC is not affected by sample imbalance. The Area Under Curve (AUC) coefficients of different gestures in the figure are close to 1, with 5 gestures having an AUC coefficient of 1 and a minimum AUC coefficient value of 0.98. From this, it can be seen that the gesture recognition model proposed in this article can achieve high recognition rates in cases of imbalanced sample distribution.

#### Model robustness verification

(a) Robustness of scenes variety

**Figure 15:**
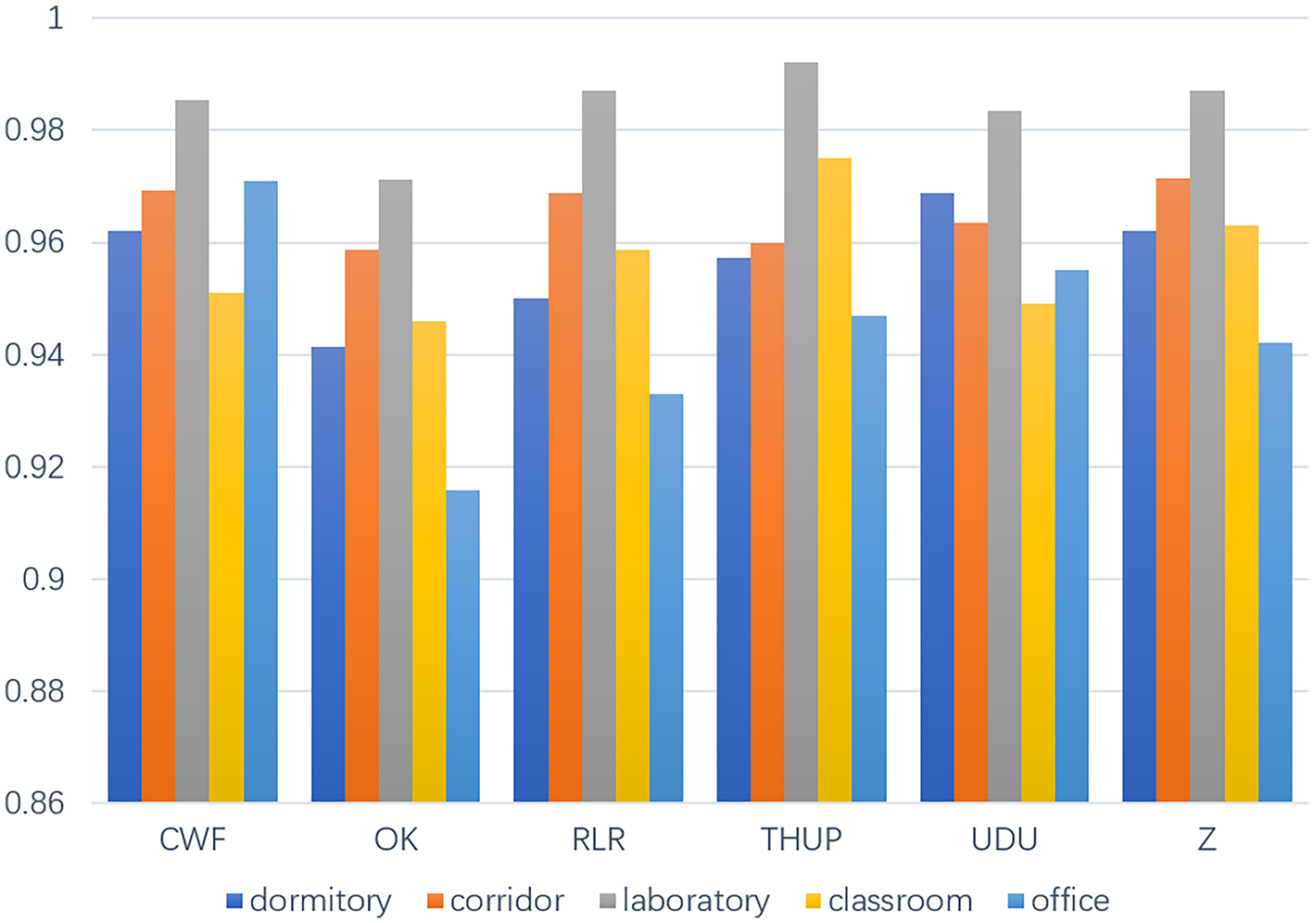
Comparison of accuracy in various scenarios.

The environment in different scenarios leads to significant differences in the reflection, absorption, and scattering of millimeter wave signals by obstacles. Therefore, the gesture signals obtained in various scenarios have different signal strength, clutter, and other factors, resulting in significant differences of gesture signals. This means that it more difficult to recognize a gesture in various scenarios. It is necessary to verify the scene generalization ability and scene robustness of the method. Experiment is implemented in laboratory, corridor, dormitory, office and classroom to verify the scene difference of the method. For every gesture in each scenario, 30 samples have been obtained. The laboratory is a scenario with large space and minimal interference factors. The corridor is a narrow space surrounded by walls and doors. Dormitory is a small space with many daily necessities and normal activities for other personnel. The class is a scenario with large space and more interference factors such as desks and chairs. The office has the similar space size with dormitory but has fewer things. CWF, OK, RLR, THUP, UDU and Z six types of gestures were selected to illustrate the impact of scene variety. As is shown in Fig. 15, The recognition results get the maximum accuracy in laboratory with 98.13%, the minimum accuracy in dormitory with 94.24%, and with a 95.36% in the corridor. Furthermore, we can get the conclusion that micro actions are more affected by the scene than macro actions. The recognition accuracy of UDU and Z with macro actions is more than 95% in each scene, however, the accuracy of OK and THUP with micro actions is less than 94% in dormitory. By comparing the recognition accuracy in three scenarios, there are relatively large difference in three scenes. The best results were achieved in laboratory with lager space and less interference. The dormitory with more interference achieved poor results.

We analyze the reasons for the differences in gesture recognition performance of variety scenarios, as well as the differences in the impact of factors such as the material, position, and motion status of obstacles and interferences on the generation of gesture signals in different scenes. In the scenes with larger space, the recognition accuracy of the laboratory is higher than that of the classroom because there are fewer interferences in the laboratory, and there are many tables, chairs, and other interferences in the classroom that generate echoes. In relatively small apartment and office scenes, the office has higher recognition accuracy due to the fewer objects placed in the office and the fact that wood is the main material, while dormitories have more living items and more metal materials, resulting in more reflected clutter and differences in recognition accuracy in different scenes.

(b) Robustness of personnel variety

**Figure 16:**
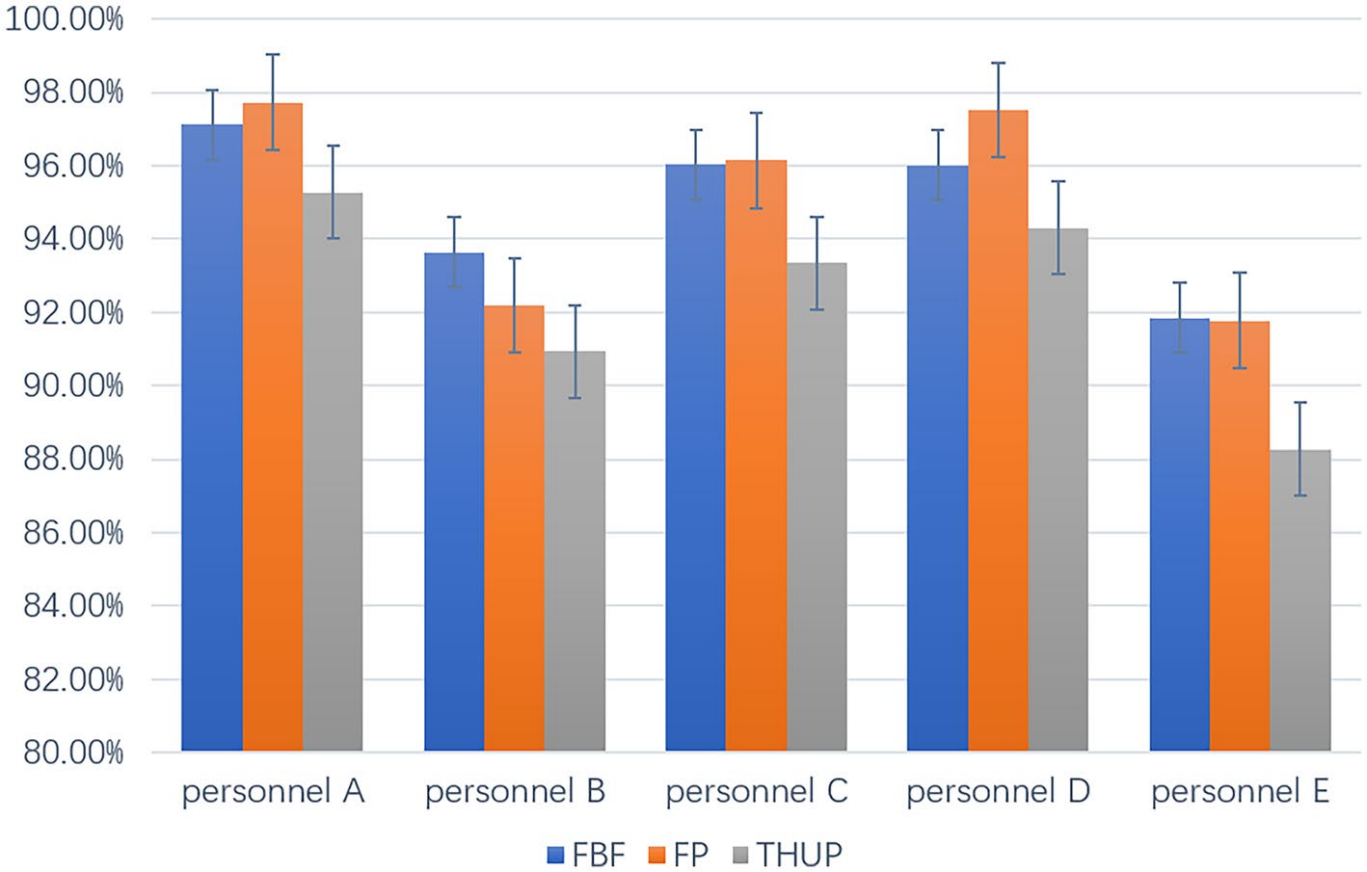
Comparison of accuracy among various personnels.

The actions of different personnels have distinct personalized characteristics, such as their body height and weight, palm size, finger lengths, action speed, etc. Whether the differences in these key features will lead to model recognition errors is a question that must be considered. In order to verify the stability of the model in recognizing gestures of different individuals, inviting 3 male participants (A, B, C) and 2 female participants (D, E) a total of 5 experimenters to the personnel variety experiment. The Physical information of experimenters is show in Table 5. As shown in Fig. 16, the recognition accuracy of different personnels is 95.30%, 90.41%, 93.55%, 93.96%, and 91.73%, respectively. Personnel A has the similar somatotype with the training data participator. The lowest accuracy of his gestures is 93.28% for SF. The accuracy of personnel B and personnel E is much lower than personnel A, The further reason is that their significant differences with training data participator. The lowest accuracy of B is 86.33% for the gesture THUP. Though personnel variety pose certain challenges to the method, it can be seen that the model has robustness for personnel variety. We analyze the differences in recognition performance among different personnels. The low recognition accuracy of personnel B and E is due to the significant difference in height between these two individuals and the training data collector, as well as the difference in radar cross-section of arm size, resulting in differences in echo signals and resulting differences in gesture features.

**Table 5:** Physical information of personnels for robustness experiment.

Personnel	A	B	C	D	E
Height (cm)	175	188	178	172	160
Weight (Kg)	65	94	64	55	48
Gender	Male	Male	Male	Female	Female

(c) Robustness of location variety

The different positions of experimental gestures relative to the radar result in different attenuation and multipath reflections of electromagnetic signals, leading to amplitude and phase differences in the received signal, which means different distances and angles, resulting in differences gesture features. The impact of feature differences on the accuracy of gesture recognition methods needs further verification. Experimental verification was conducted on the gesture recognition performance at different distances and angles, with verification distances of 0.4 m, 0.8 m, 1.2 m, and 1.5 m, and verification angles of 0°, 30°, 60°, and 90°, respectively. A total of 16 experimental positions were designed. Collect 20 experimental data for each gesture at 16 experimental locations. The verification results are shown in Table 6.

**Table 6:** Accuracy of different distances and angles.

	0.4 m	0.8 m	1.2 m	1.5 m
90°	97.65%	97.20%	95.44%	91.13%
60°	95.56%	94.78%	94.15%	90.32%
30°	92.13%	91.62%	90.49%	86.46%
0°	87.47%	86.55%	85.13%	84.01%

From Table 5, it can be seen that the recognition accuracy varies at different positions, with an overall recognition rate distribution of 84.01–97.65%. At positions 90° and 0.4 m, the recognition accuracy is highest at 98.97%, while at positions 0° and 1.5 m, the recognition accuracy is lowest at 84.01%. At the same angle, as the distance increases, the recognition accuracy decreases. At the same distance, as the angle increases from 0° to 90°, the recognition accuracy increases.

**Figure 17:**
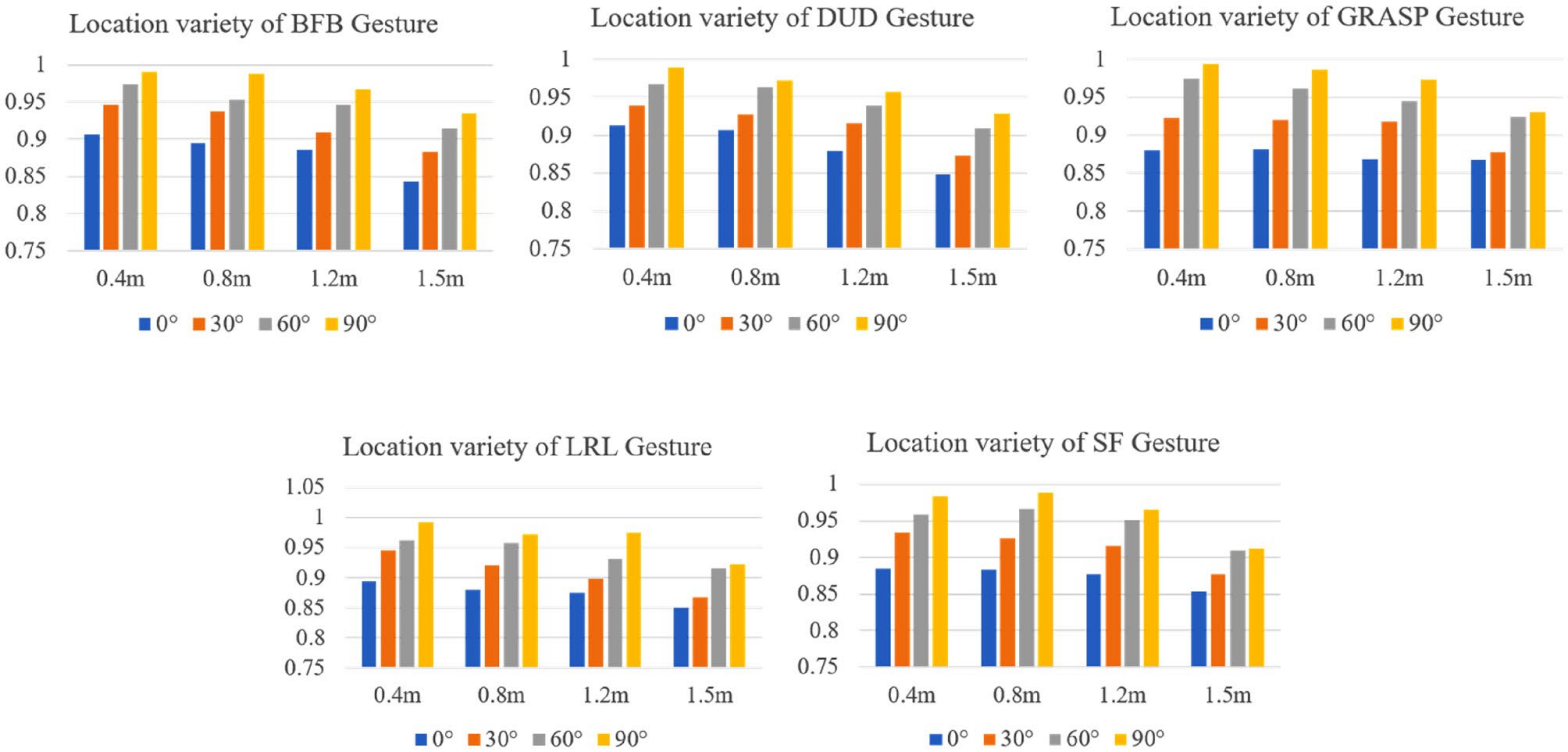
Comparison of accuracy in various locations.

As shown in Fig. 17, the same gesture action at different angles will produce different cutting angles to the radar electromagnetic wave. Thus, different echo signals are generated, resulting in different characteristics. For example, the motion directions of the same gesture at 0° and 90° angles are perpendicular to each other, which means that the same gesture has different motion directions compared with radar. At different distances, the same gesture and radar signal have different cutting surfaces, resulting in different echo signals. In order to verify the stability of the gesture recognition method proposed in this paper on the difference between direction and angle, six kinds of gestures including BFB, dud, FP, grasp, LRL and SF with multiple motion directions are selected, and compare the accuracy of each gesture recognition at different experimental locations.

It can be seen in Fig. 16 that there are certain differences in the recognition accuracy of each gesture at different positions, mainly due to the different echo signals generated by gestures on radar electromagnetic waves at different angles and distances. The effects of different directions of gesture movement are not entirely the same. Like the BFB gesture, at a 90° position, the palm is completely in contact with the radar electromagnetic wave, creating a larger reflection surface and generating a higher intensity intermediate frequency signal. At the 0° position, the contact area between the palm and the radar electromagnetic wave decreases, resulting in a lower intensity of intermediate frequency signals. The DUD gesture mainly involves hand movements up and down, and the influence of different angle gestures on the reflection changes of electromagnetic waves is relatively small. Therefore, the recognition accuracy of DUD at 0° position is higher than that of BFB, which is 87.41% and 85.76%, respectively. The GRASP and SF gestures are mainly micro movements of the fingers. At the 0° position, some subtle gestures do not have a clear perception of electromagnetic waves, resulting in lower recognition rates of 85.23% and 84.37%, respectively.

The change in distance also has a certain impact on the accuracy of gesture recognition. The gesture most affected by distance is SF, which is 94.63% at a distance of 0.4 m and 88.64% at a distance of 1.5 m, with a difference of 6 percentage points. The LRL is the least affected by distance, which is 94.07% at a distance of 0.4 m and 89.71% at a distance of 1.5 m, with a difference of 4 percentage points. The average recognition accuracy of the six experimental gestures is higher than 92%. When the distance is 1.5 m, the recognition accuracy significantly decreases, ranging from 87.85 to 88.16%. It can be seen that the method proposed in this article is robust to location variety.

From the experimental results, it can be seen that there are significant differences in recognition accuracy at different angles and distances. The reasons for the differences are analyzed as follows. The viewing field of IWR1643 radar is − 60°– + 60°. In the 0° direction, when the gesture is outside the field of view, the gesture echo signal mainly comes from the multipath reflection of radar signals, resulting in significant differences between gesture features and training data features. At a distance of 1.5 m, the arm receives a lower intensity and energy of the radar signal, resulting in a lower intensity and greater energy loss of the excited echo signal. The radar receives a lower intensity of the echo signal, resulting in less obvious features.

(d) Robustness of velocity variety

**Figure 18:**
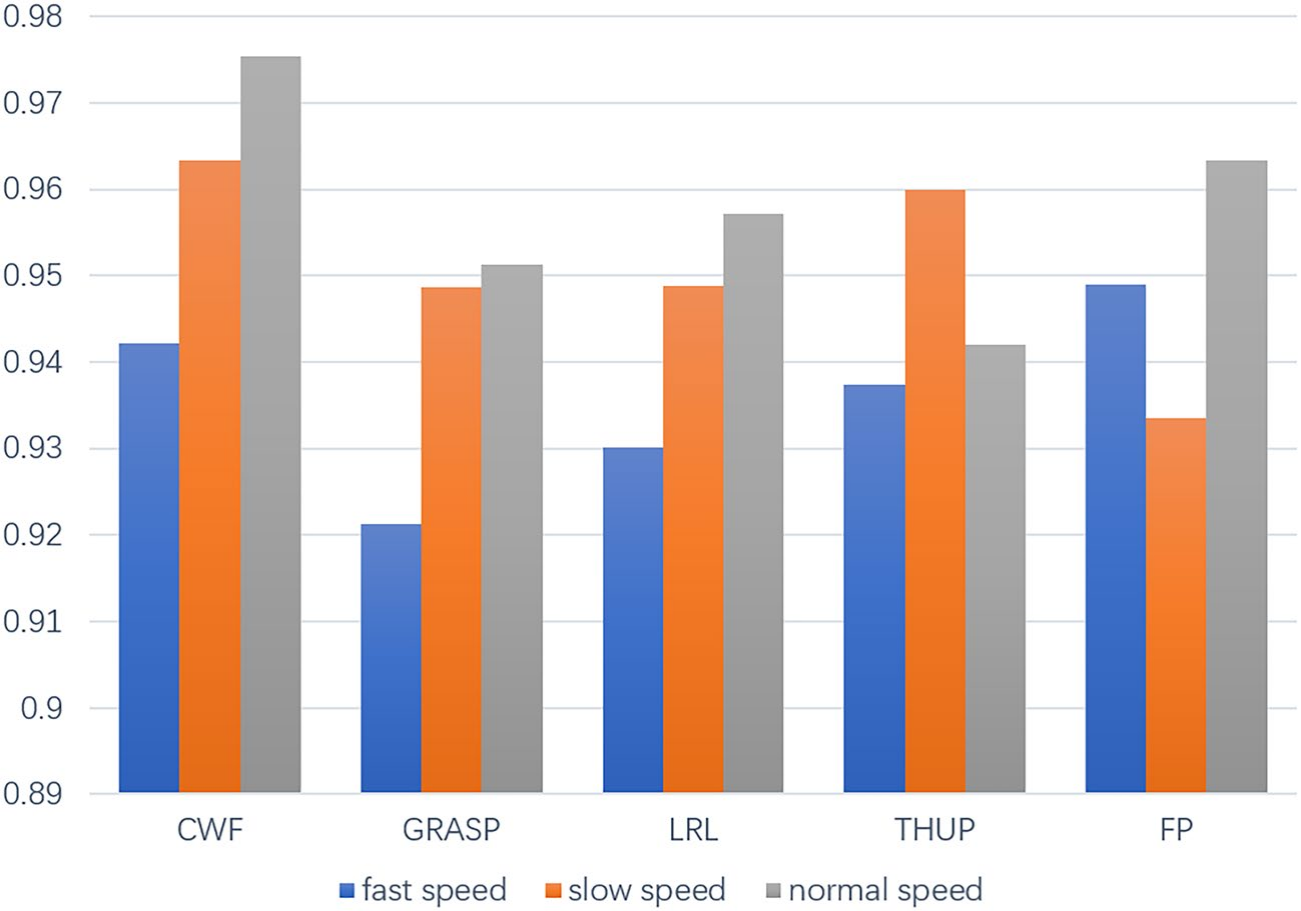
Comparison of accuracy of various speed.

Speed is an important perception factor for radar to target gestures, and the same gesture with different speeds will produce different velocity features. Whether these feature differences will cause the recognition performance of the model to deteriorate needs further verification. In order to verify the robustness of the model for recognizing gestures at different speeds, the training data collection speed (32 frames) was used as the standard speed to verify three different speeds of gestures, namely fast gestures (16 frames), standard speed gestures (32 frames), and slow speed gestures (64 frames). 30 sets of data were collected for each experimental gesture at three different speeds. The comparison of recognition effects of CWF, GRASP, THUP, LRL, and FP is shown in the figure. The result of this experiment is show as Fig. 18. The accuracy of fast gesture recognition is 94.03%, the accuracy of normal speed gesture recognition is 96.59%, and the accuracy of slow speed gesture recognition is 95.17%. The accuracy of normal speed gesture recognition is the highest, CWF, GRASP, and LRL has the accuracy of 96.33%, 94.87%, 94.88%, respectively. However, the accuracy of fast gesture recognition is lower than that of slow speed gesture recognition. In fast state, the accuracy of THUP and GRASP gesture recognition is the lowest, with 93.73% and 92.13%, respectively. The FP gesture has the highest accuracy of 94.89%. In slow speed, CWF and FP have the highest recognition accuracy, with 97.54% and 96.34%, and the lowest accuracy gesture is THUP with recognition accuracy of 94.2%. The reason behind this is that when the target gesture moves at a fast speed and approaches the velocity resolution, it will cause confusion in velocity characteristics, resulting in loss of recognition results. However, the velocity resolution configured by the experimental radar in this article is 4.87 cm/s. When a gesture is completed within 16 frames, it means that the gesture time does not exceed 0.5 s, and the distinguishable velocity difference is 2.435 cm/s. When gestures with more micro actions such as GRASP are in a fast state, it will cause confusion in velocity characteristics of different hand positions.

## Conclusion

This paper proposes a multi feature fusion gesture recognition method based on millimeter wave radar for the recognition of millimeter wave gestures in complex scenes. Firstly, collecting radar data in four different scenarios enhances data diversity and better reflects scene complexity. Secondly, filter and denoise gesture data to improve signal-to-noise ratio. Extract RTM, DTM, and ATM feature maps to fully represent the motion states of gestures such as distance, speed, angle, and timing, and analyze the feature maps in detail. Finally, a lightweight Multi-CNN-LSTM neural network model was proposed, achieving high recognition accuracy. This article compares the recognition effects of different features and verifies the correctness of feature extraction and the necessity of feature fusion. We compared the recognition accuracy and memory requirements of seven different neural network models. The experimental results show that the proposed Multi- CNN-LSTM method has slightly better recognition accuracy than other models, and the memory requirement is not the highest. The proposed model has been robust in terms of personnel, position, angle, speed, scene, and other aspects. This indicates that the method has good performance in recognition accuracy, portability, and robustness. The research in this article advances the application of gesture recognition in human–computer interaction scenarios such as smart homes, sign language communication, and games. However, there are still some issues that need further research. This article only considers single gesture recognition in complex scenes within the sight range. Therefore, gesture recognition in non-sight range scene will be a future research work. In addition, as the most common gesture form, continuous gesture recognition based on multi-feature fusion is another research direction, which involves accurate segmentation and feature extraction of continuous gestures.

## Data Availability

The datasets generated during and/or analyzed during the current study are not publicly available due to [Data and processing information related to product development] but are available from the corresponding author on reasonable request.
